# Supplementary material for: DeepHiC: A generative adversarial network for enhancing Hi-C data resolution
Source: PLoS Comput Biol. 2020 Feb 21;16(2):e1007287. doi: 10.1371/journal.pcbi.1007287 (PMC7055922; doi:10.1371/journal.pcbi.1007287)
Supplement: S1 Note — (PDF) [file pcbi.1007287.s001.pdf]

Supplementary Note S1. The setting of colorbar for heatmaps plotting.

Adjustments in colorbar would affect the visual effect of heatmaps. Here we give the description of how we used colorbar setting for different Hi-C contact maps. First, we post our python code for plotting heatmaps as follows,

```
import numpy as np
import matplotlib.pyplot as plt
import matplotlib.cm as cm

mat # the Hi-C matrix will be plotted
# the bottom (low) and top (high) values are determined by two ways
# 1. low = min(mat), high = max(mat)
# 2. low = min(mat), high = the 99-th percentile of mat
plt.imshow(mat, cmap=cm.Reds, vmin=low, vmax=high)
```

All heatmaps in comparison analyses used the same "cm.Reds" as the colormap. As for the low and high variables for plotting heatmaps, there are two options for different Hi-C contact maps.

Option 1: for DeepHiC-enhanced, HiCPlus-enhance, HiCNN-enhanced matrices, the colorbar ranges from the [min, max] value of each matrices. Because DeepHiC outputs Hi-C matrix with values range from 0-1, and HiCPlus/HiCNN outputs Hi-C matrix with values range from 0-100. There is no need for adjusting colorbars for them.

Option 2. for original, downsampled, Boost-HiC-hanced matrix. Because they still contain a small part of outlier with extremely high value. We filtered out the outliers with a cut-off of 99-th percentile to ensure equality for all sub-regions.
