# Supplementary material for: DeepHiC: A generative adversarial network for enhancing Hi-C data resolution
Source: PLoS Comput Biol. 2020 Feb 21;16(2):e1007287. doi: 10.1371/journal.pcbi.1007287 (PMC7055922; doi:10.1371/journal.pcbi.1007287)
Supplement: S2 Note — (PDF) [file pcbi.1007287.s002.pdf]

A guide of choosing trained models of DeepHiC

Translating the downsampling factors to read coverage or data distribution is indispensable for researchers/users. Considering that there is no concrete criterion of 10-kb Hi-C data, the strategy of choosing model should be related to the training data we used. The detailed answer is listed in the following:

1. **Users could choose the model according to the distribution of their Hi-C matrix**, especially focus on the values of top 95-99.9 percentiles (in all non-zero values). As shown in Figure A-a in this note, for different downsampled data, the cutoff values for low-coverage input (*-lrc* parameter for our code in GitHub) were close to the 99.9 percentile values in our trained data. For a low-coverage data, user should use the model where their 99.9-percentile value is close to or lower than the cutoff value of chosen model. A caveat is that user should not use 25 as a cutoff value while the 99.9 percentile of their data is 100. Therefore, the model for 1/100 downsampled data is not always the best choice.

2. **If user has a Hi-C data which is latently in 1/16 to GM12878 data, it should be better to use the model trained on 1/16 downsampled data.** We downsampled GM12878 data to 1/25, 1/36 ratio, but still using the model trained in 1/16 downsampled data. These results have been shown in Fig. S16. Our model still worked well but performance decayed.

3. **Another important metric is the non-sparsity of input data must greater than ~10%.** Since our low-coverage input is still binned at 10-kb, the non-sparsity (#non-zeros/#all-pairs) of input decreased when the downsampled factor increased, as shown in Figure A-b in this note. That means for 1/100 downsampled data (non-sparsity=9%), our model imputes ~75% cells from zeros to fit the original data (non-sparsity=84%). A lower non-sparsity of input data reaches the lower bound of our current model. Thus, prediction in 1/100 downsampled data of K562 and IMR90 didn't worked because their 1/100 downsampled data are in lower non-sparsity (Figure A-c in this note)

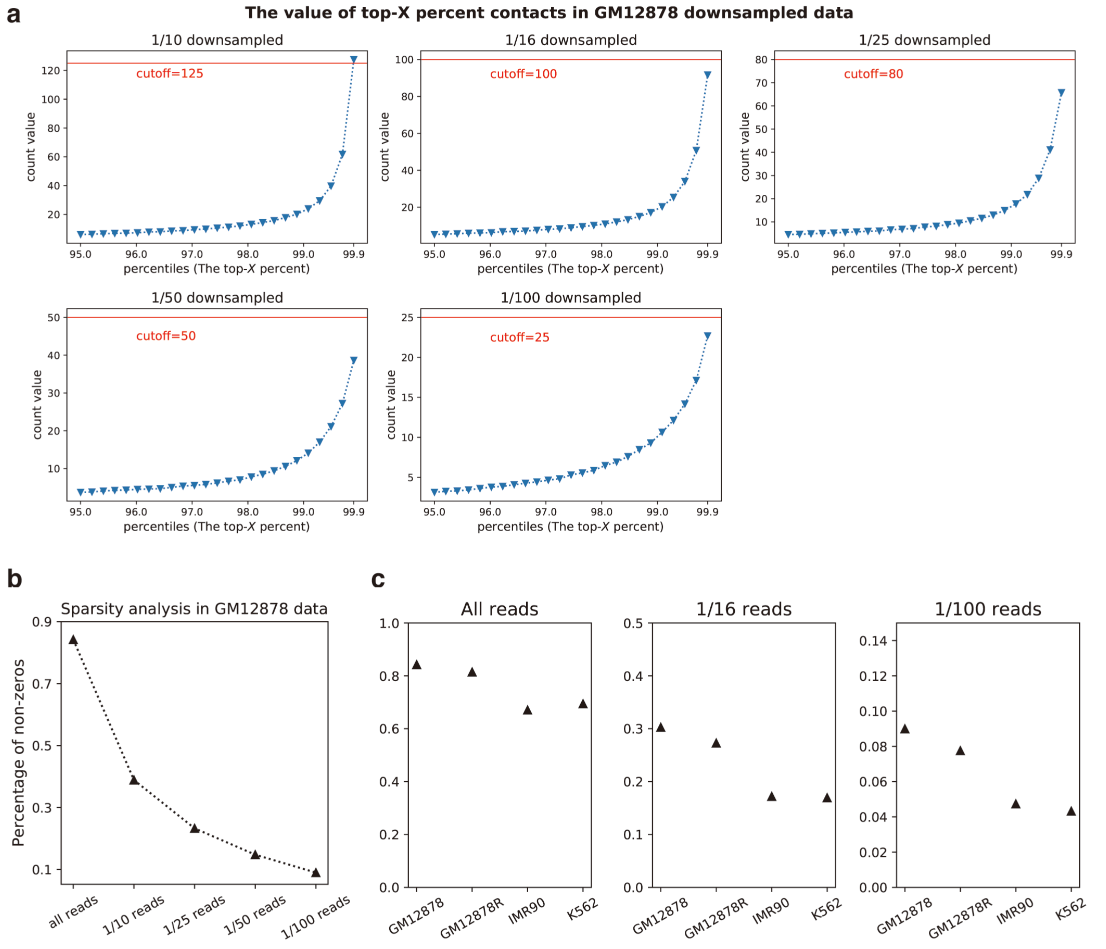

Figure. A Legend: a) The trend of top 95-99.9 percentile non-zero value in GM12878 downsampled data. The cutoff value is used for model input. **b)** The non-sparsity of training data in different downsampling factors. **c)** The non-sparsity of IMR90 and K562 data in different downsampling factors.
