# Supplementary figures and images for: DeepHiC: A generative adversarial network for enhancing Hi-C data resolution
Source: PLoS Comput Biol. 2020 Feb 21;16(2):e1007287. doi: 10.1371/journal.pcbi.1007287 (PMC7055922; doi:10.1371/journal.pcbi.1007287)

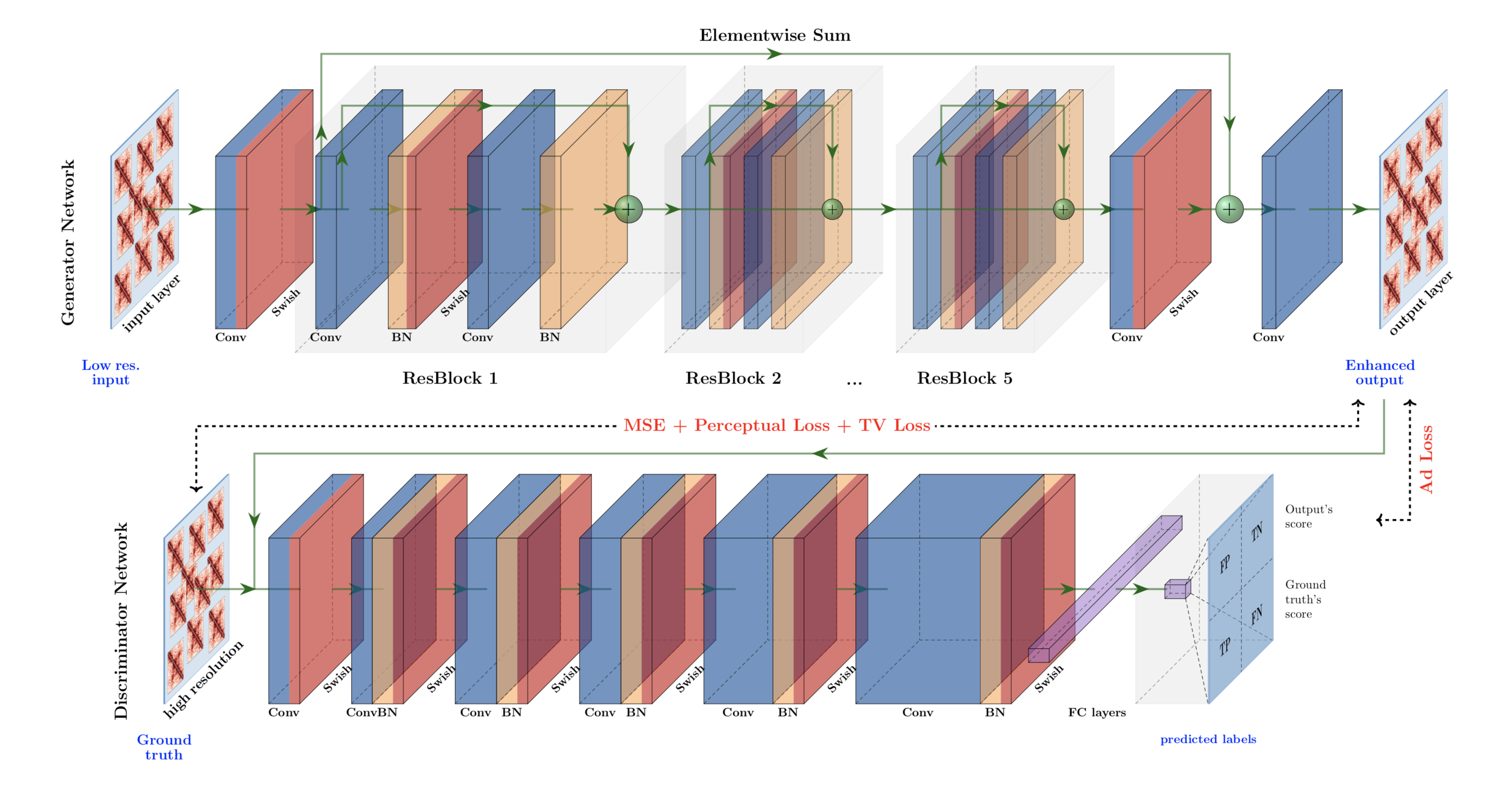

Supplement: S1 Fig — DeepHiC is a conditional generative adversarial network (cGAN) that contains two separated networks. The generator net (first row) takes low-resolution samples as input and generates the enhanced output. The discriminator net (second row), employed only during the training stage, discriminates real and generated high-resolution data. The objective function of the generator net is the sum of four loss functions: mean squared error (MSE) loss, perceptual loss, total variation (TV) loss, and adversarial (AD) loss, all of which are introduced according to the GAN paradigm. The settings used for convolution layers (blue blocks) are listed in detail in S1 Table and S2 Table. (TIFF) [file pcbi.1007287.s003.tiff]

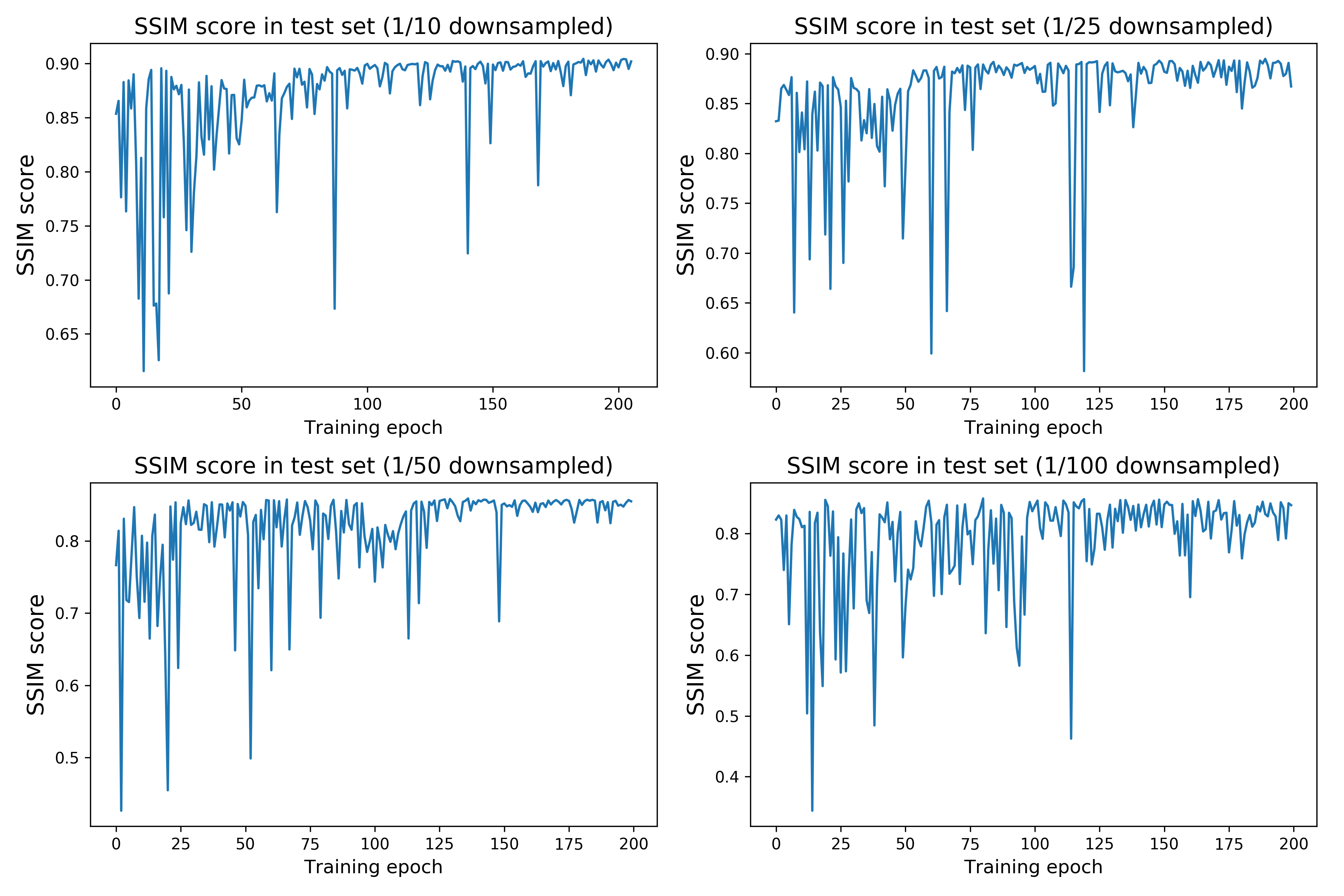

Supplement: S2 Fig — the SSIM scores in test set increased gradually and converged during the training process. (TIFF) [file pcbi.1007287.s004.tiff]

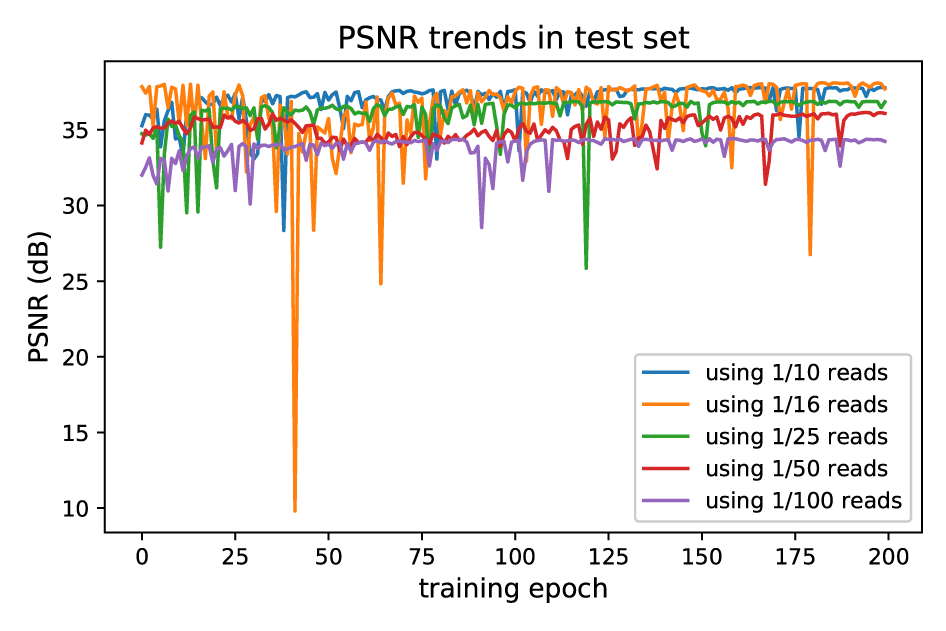

Supplement: S3 Fig — The PSNR is derived from MSE, It equals 10 × log10(1/MSE) in our experiment. (TIFF) [file pcbi.1007287.s005.tiff]

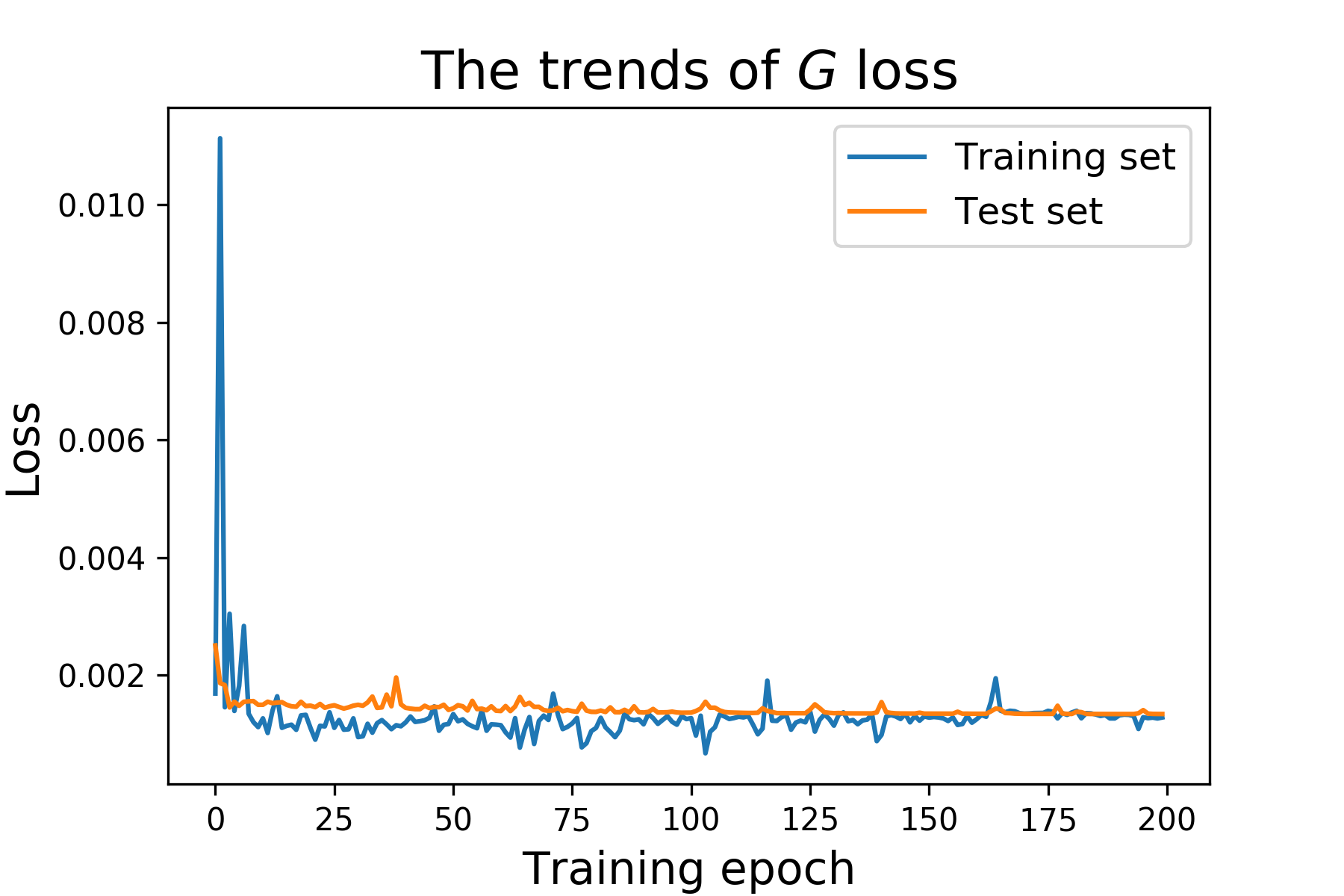

Supplement: S4 Fig — To study the possible over-fitting issue in our model, we calculated the generator loss (G loss) during the training process on the training sets (chromosome 1–14) and test sets (chromosome 15–22) in GM12878 cell type. We observe that the loss in training and test sets keep the same trend in the entire training process. (test perform on 1/25 downsampling data) (TIFF) [file pcbi.1007287.s006.tiff]

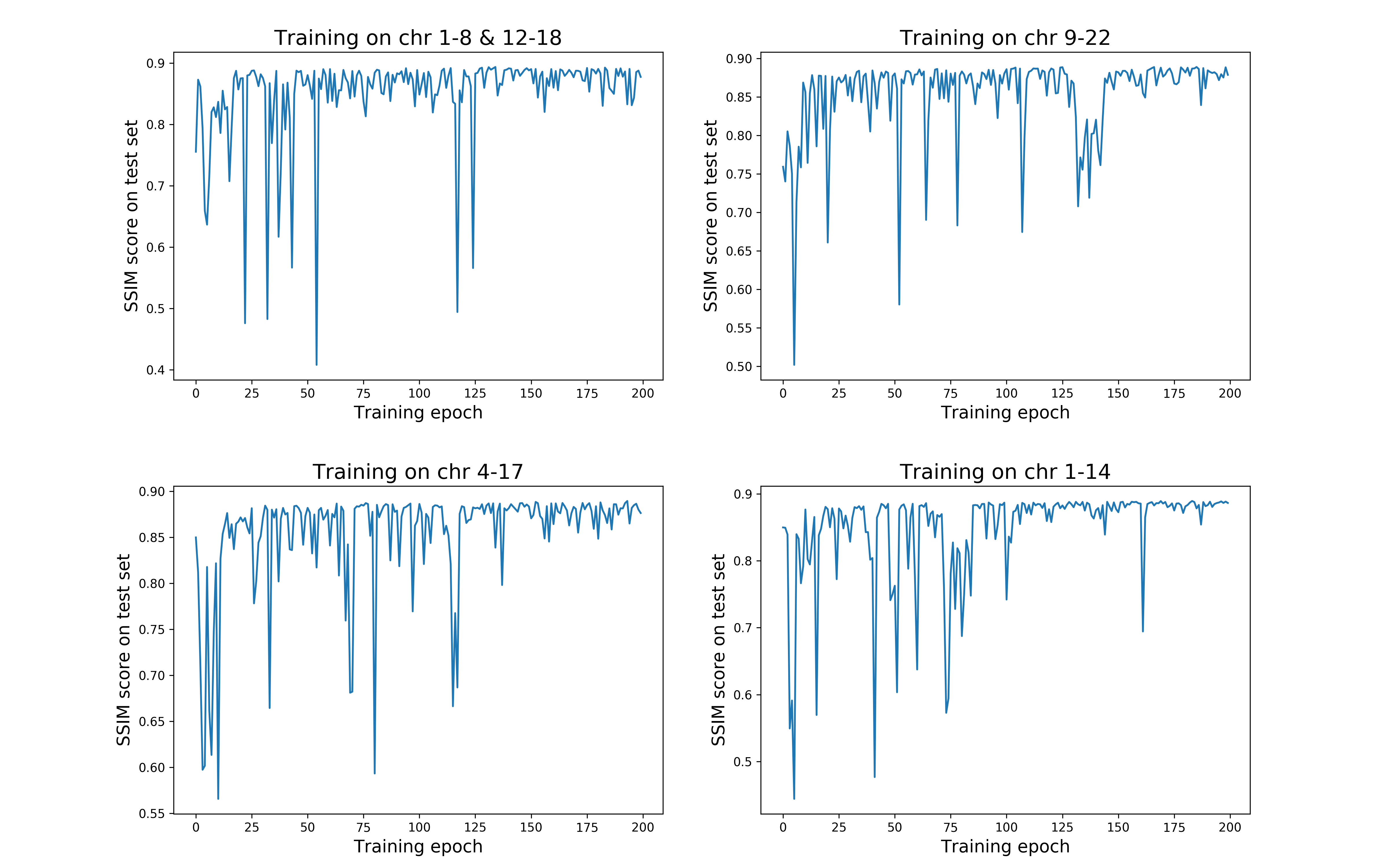

Supplement: S5 Fig — Training on different chromosomes in the GM12878 dataset. SSIM scores are evaluated in remaining chromosomes in GM12878 dataset besides training set. (test perform on 1/25 downsampling data) (TIFF) [file pcbi.1007287.s007.tiff]

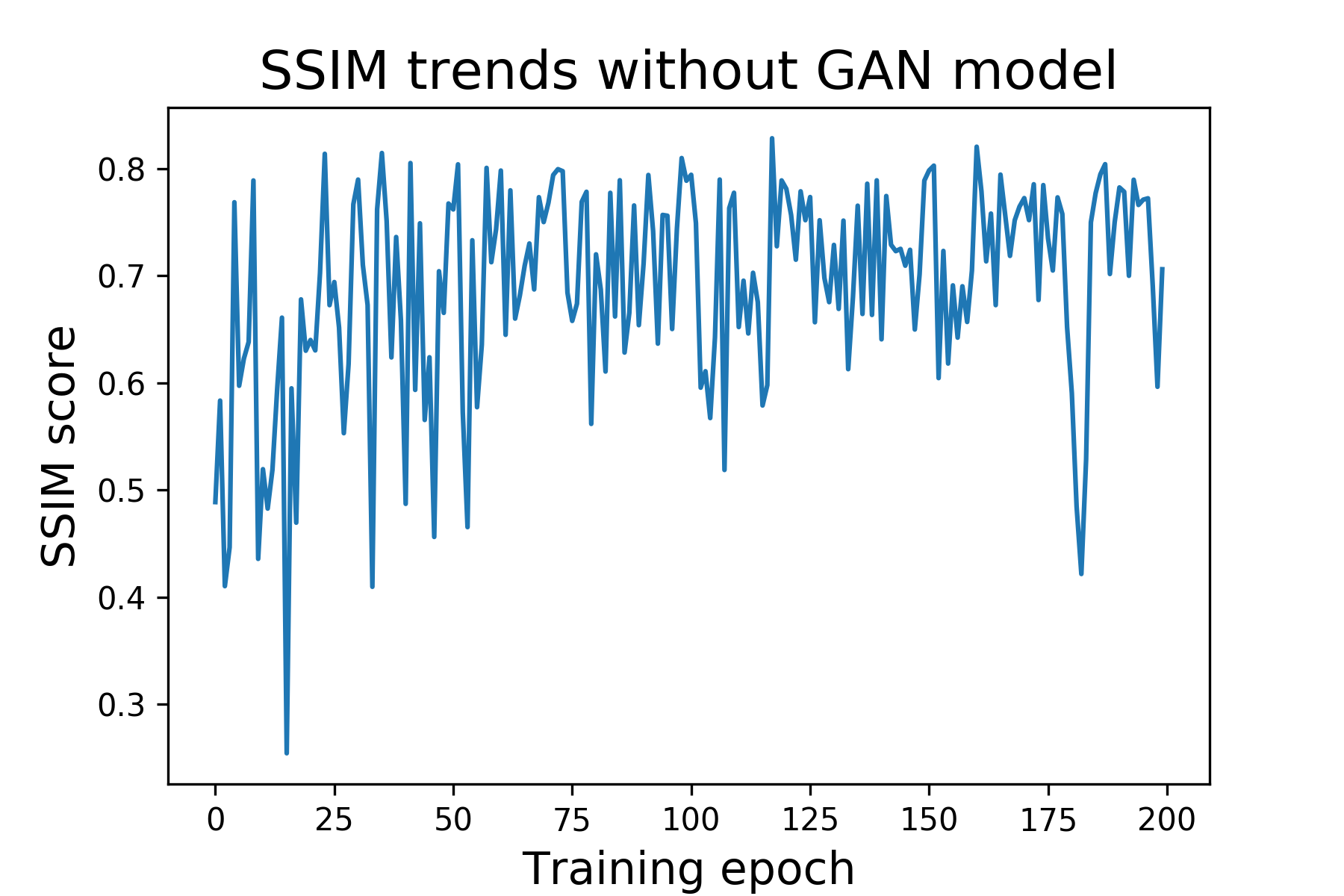

Supplement: S6 Fig — The SSIM scores in test set did not converge when training the generator net without pitting against the discriminator net. (TIFF) [file pcbi.1007287.s008.tiff]

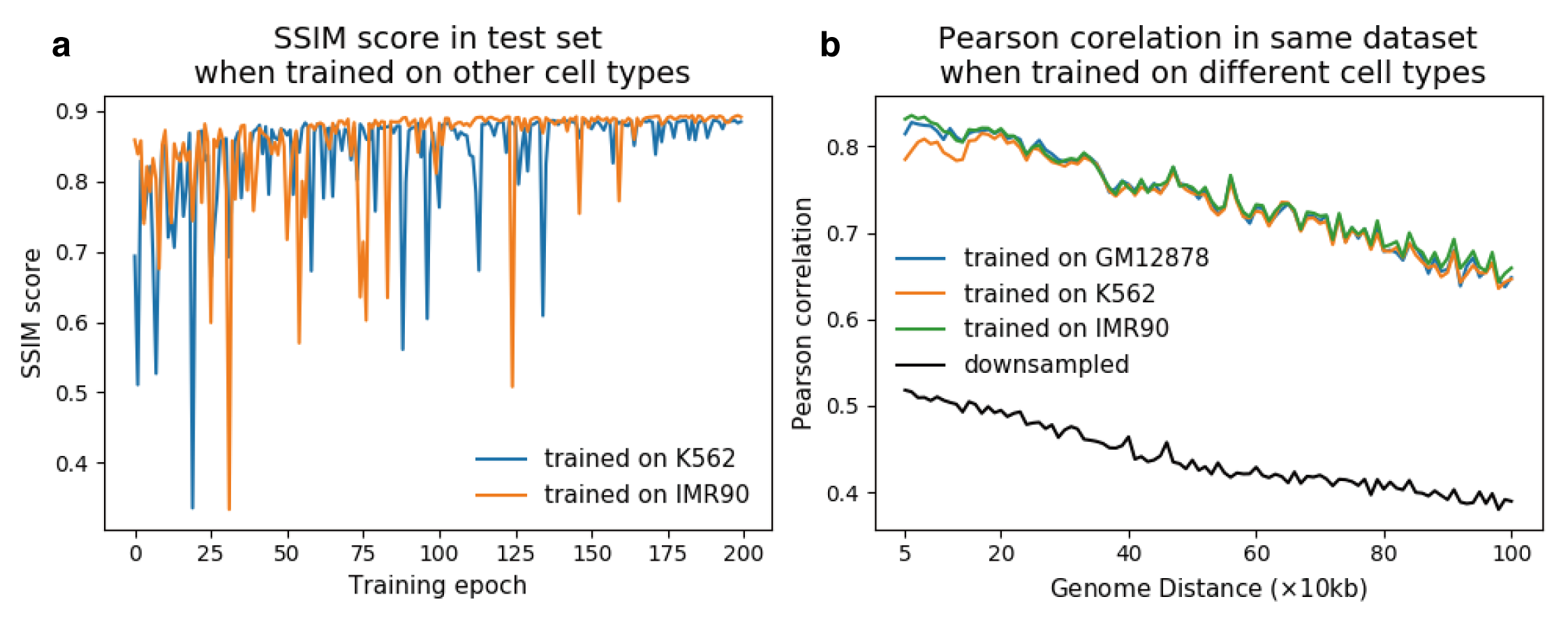

Supplement: S7 Fig — a) The SSIM scores in test set (chr15-chrX) are approaching nearly 0.9 when training DeepHiC on chr1-chr14 data in the K562 or IMR90 cell line. b) Pearson correlation in the same set (chr15-chrX in IMR90) when using trained model from different cell type. (TIFF) [file pcbi.1007287.s009.tiff]

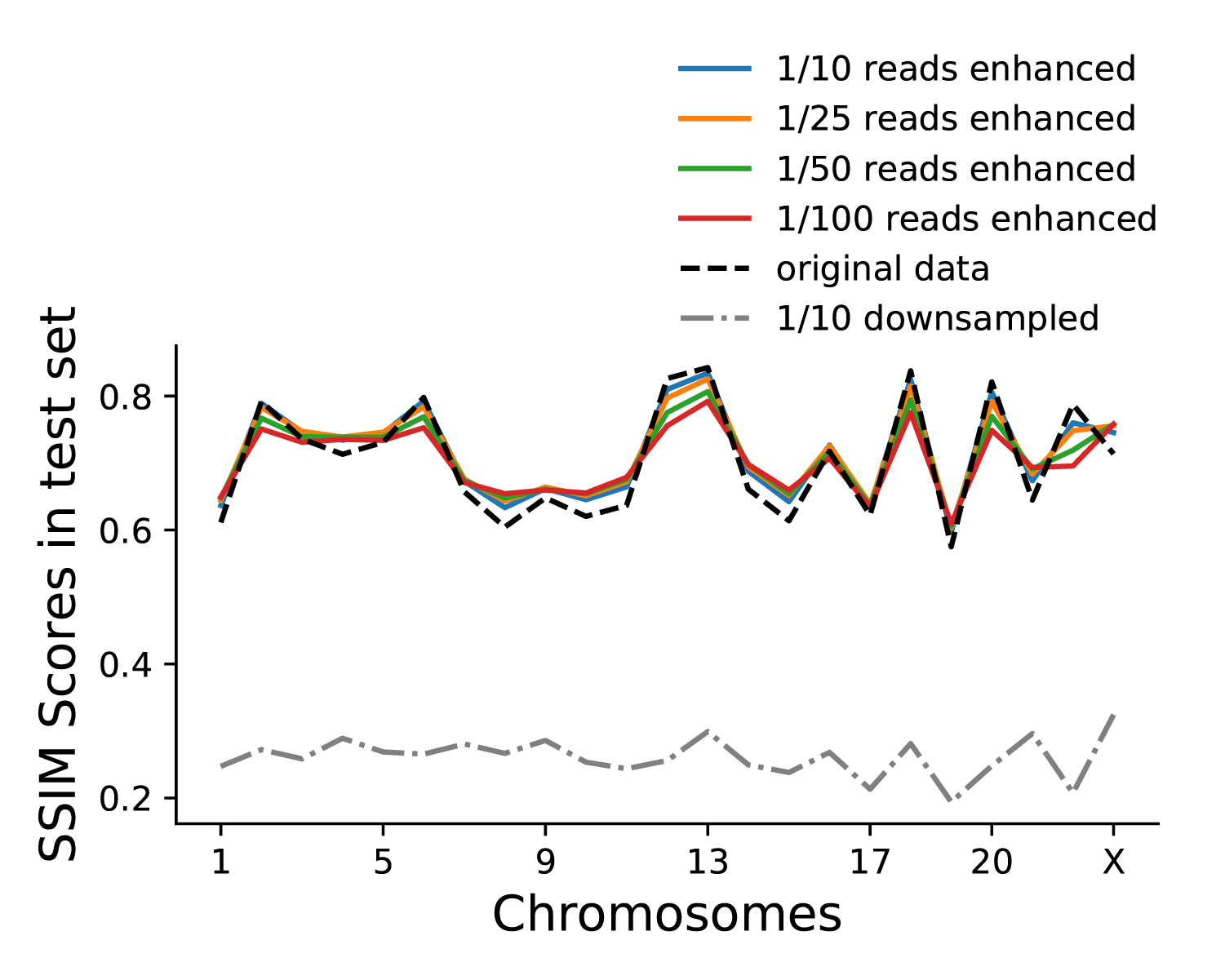

Supplement: S8 Fig — After training DeepHiC, we evaluate the performance of DeepHiC in enhancing low-resolution data derived from different downsampled ratios. Comparing with the replicated assay in GM12878 cell line, DeepHiC achieved comparative performance in SSIM scores in both training and test set at each downsampling ratio. (TIFF) [file pcbi.1007287.s010.tiff]

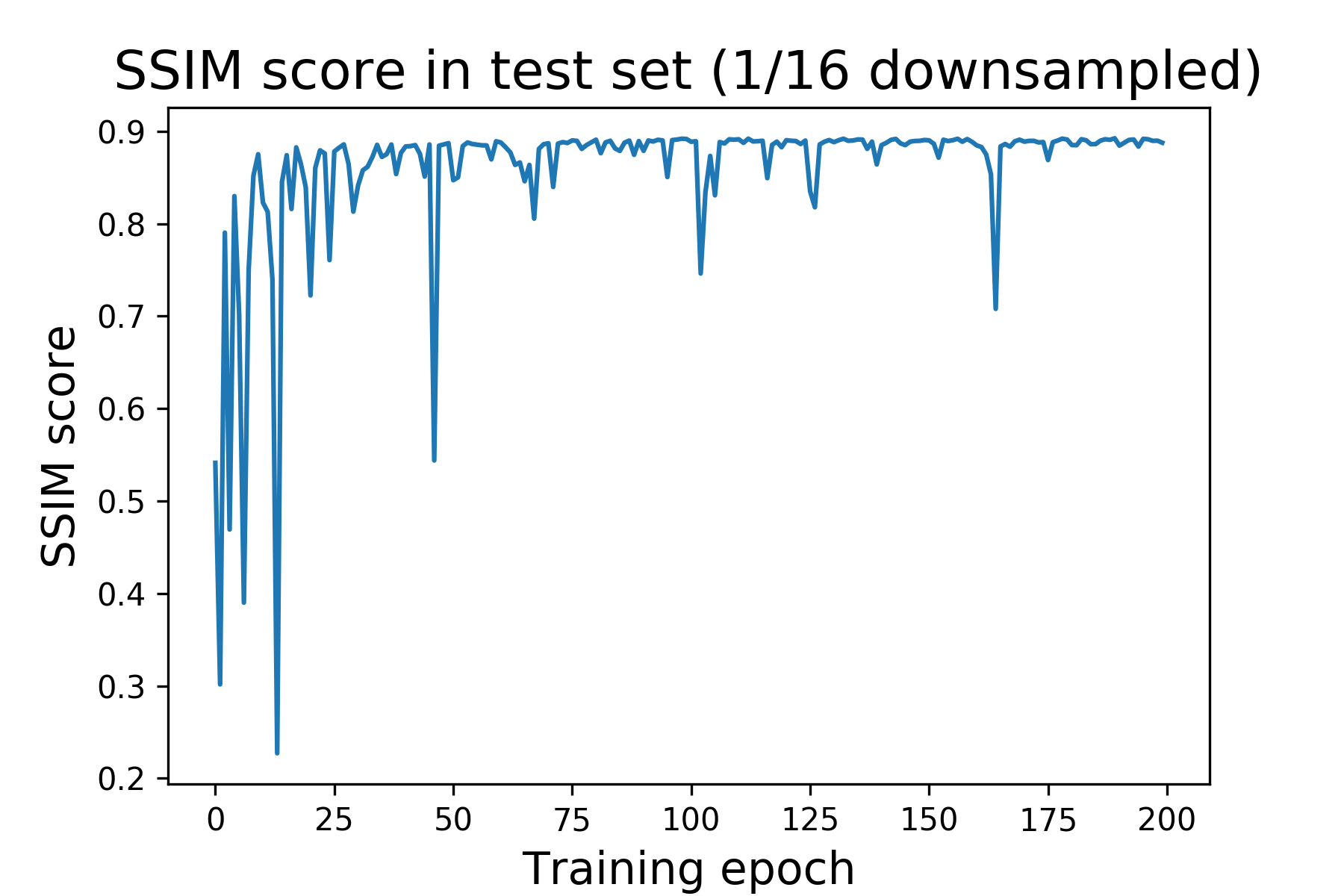

Supplement: S9 Fig — We randomly downsampled the original reads to a 1:16 ratio as low-resolution input, then trained DeepHiC on chromosomes 1–14 and tested it on chromosomes 15–22 (i.e., test set), in GM12878 cell line. As training progressed, the structure similarity index (SSIM) between predicted and real high-resolution data gradually increased and converged on the summit value of 0.9. (TIFF) [file pcbi.1007287.s011.tiff]

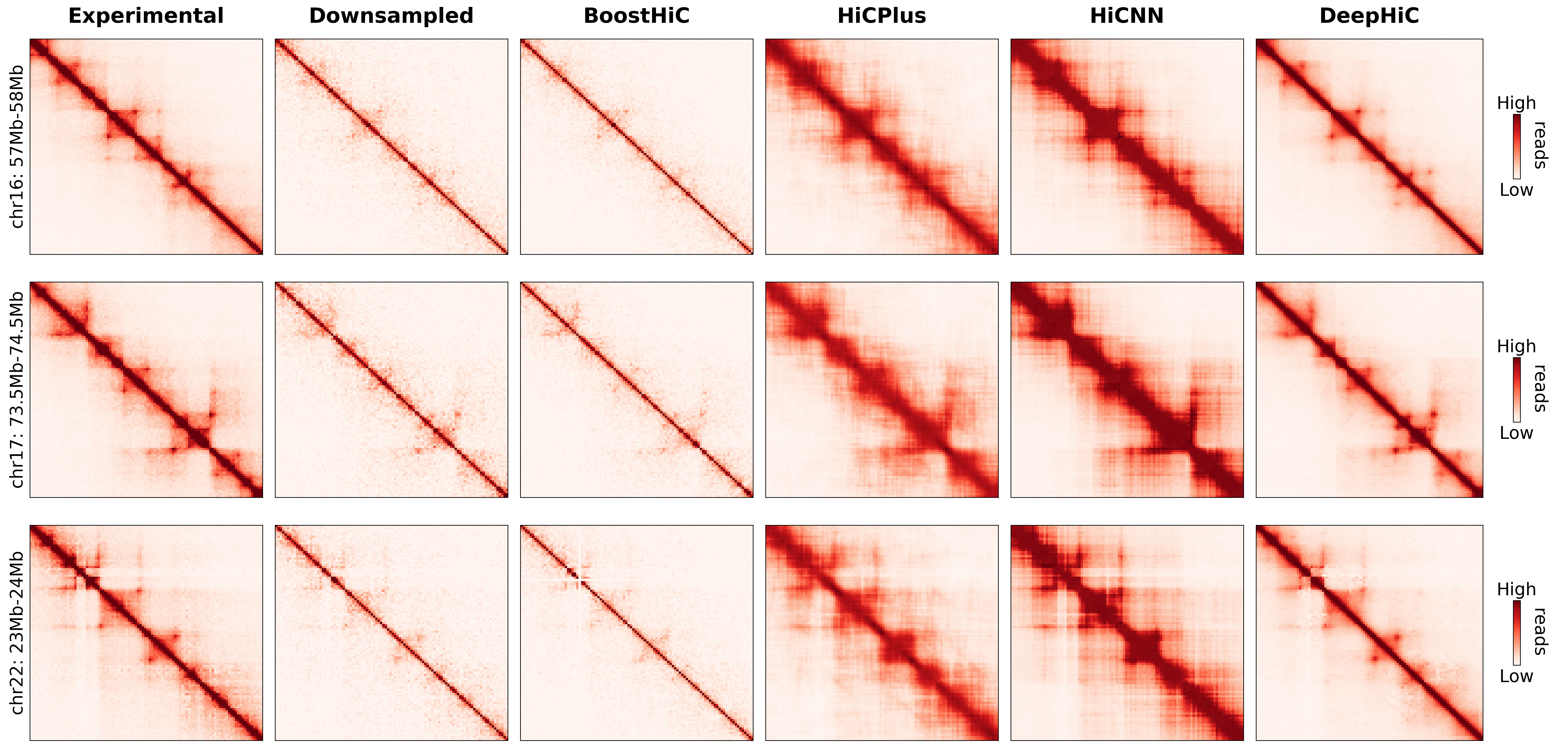

Supplement: S10 Fig — Here we present the heatmap of three 1 Mb × 1 Mb (100 bins) sub-regions extracted from chromosomes 16, 17, and 22 from the replicate assay of GM12878 cell line. Colorbar setting: S1 Note. (TIFF) [file pcbi.1007287.s012.tiff]

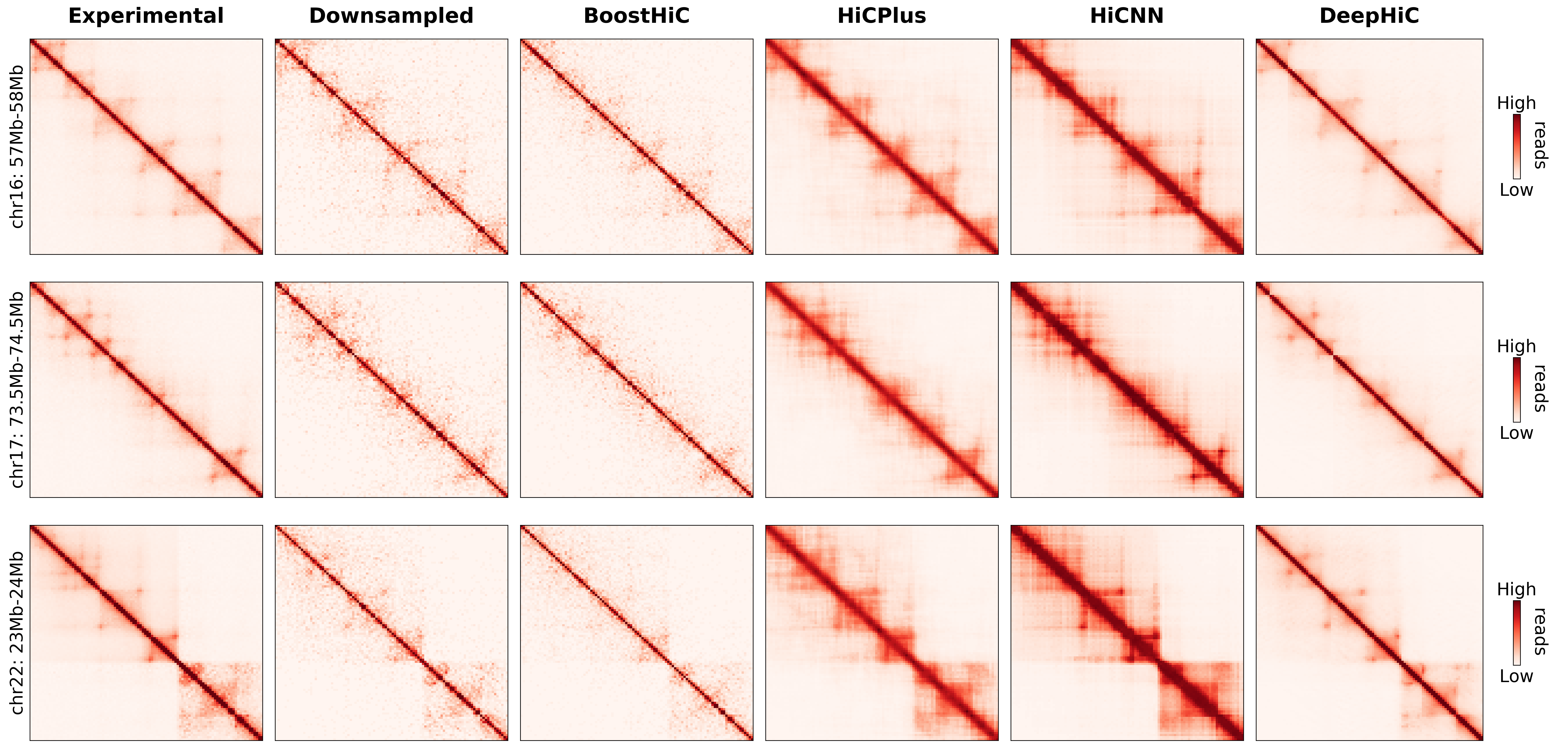

Supplement: S11 Fig — Here we present the heatmap of three 1 Mb × 1 Mb (100 bins) sub-regions extracted from chromosomes 16, 17, and 22 from the K562 cell line. Colorbar setting: S1 Note. (TIFF) [file pcbi.1007287.s013.tiff]

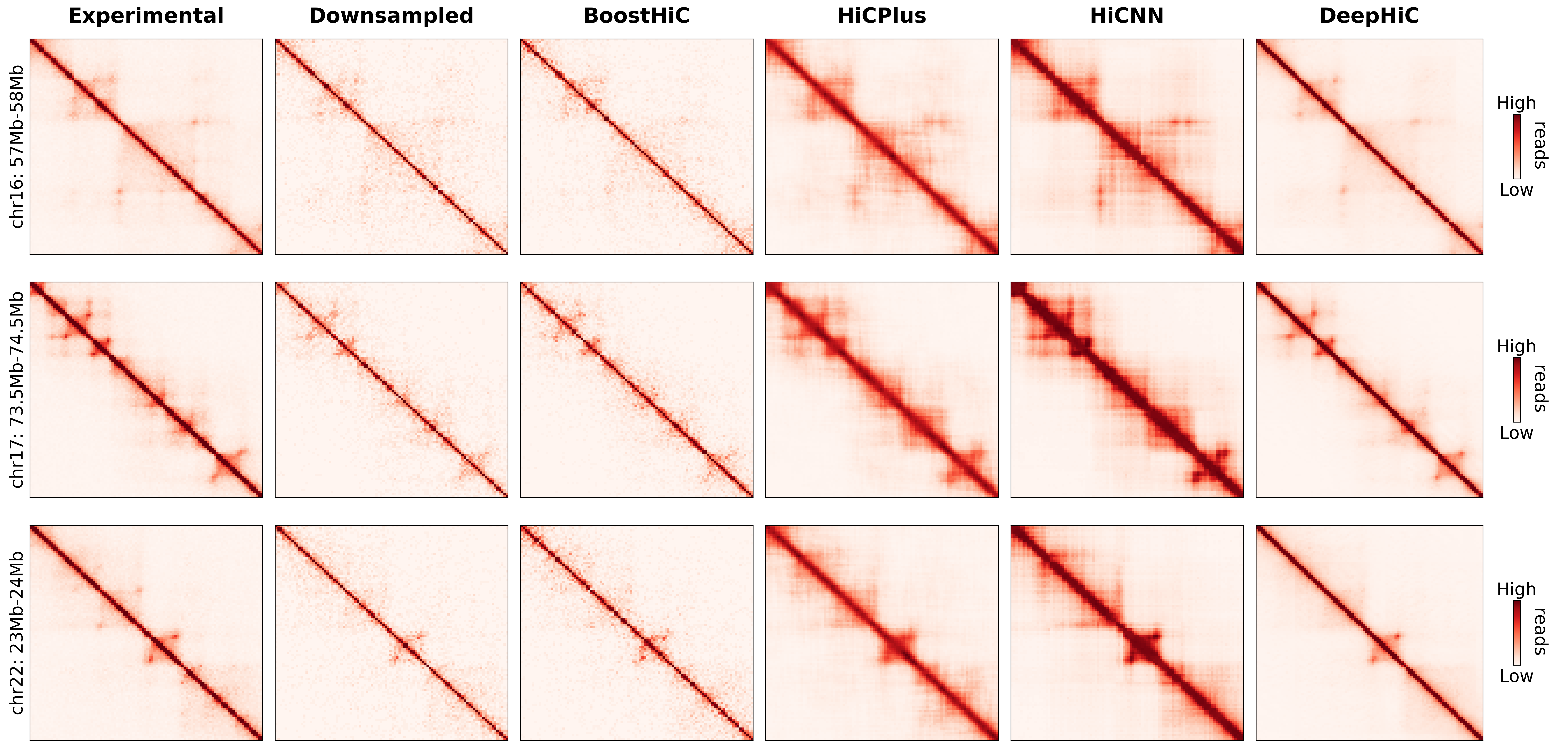

Supplement: S12 Fig — Here we present the heatmap of three 1 Mb × 1 Mb (100 bins) sub-regions extracted from chromosomes 16, 17, and 22 from the MR90 cell line. Colorbar setting: S1 Note. (TIFF) [file pcbi.1007287.s014.tiff]

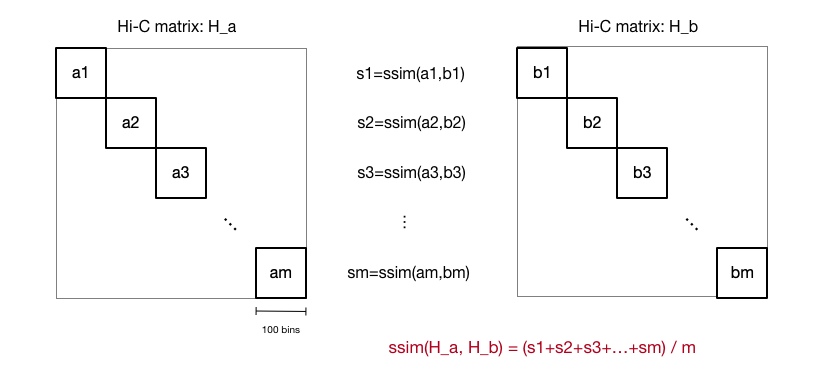

Supplement: S13 Fig — In our experiments, we calculated SSIM of the 1Mb x 1Mb (100 bins x 100 bins) sub-regions at the diagonal as those regions cover the genome distance of interest. We calculate the mean of SSIMs between all 1Mb x 1Mb sub-regions with non-overlap at the diagonal across the entire genome to be the final SSIM score between two large Hi-C contact maps. (TIFF) [file pcbi.1007287.s015.tiff]

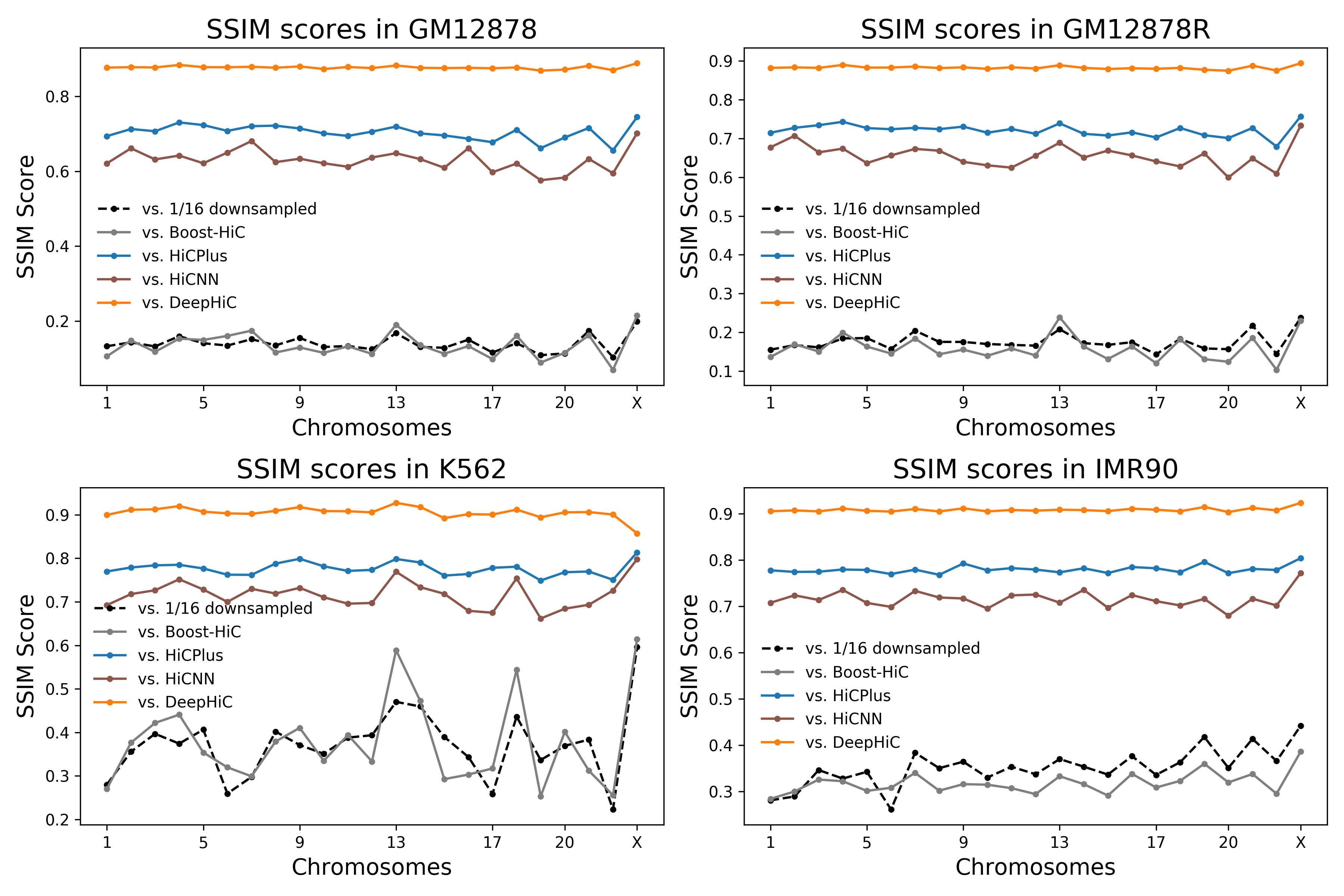

Supplement: S14 Fig — Between three types of non-experimental data: 1/16 downsampled, HiCPlus/Boost-HiC/HiCNN-enhanced, and DeepHiC-enhanced data in various cell types. We calculated the SSIM scores of three non-experimental data, as compare to real high-resolution data for all chromosomes. (TIFF) [file pcbi.1007287.s016.tiff]

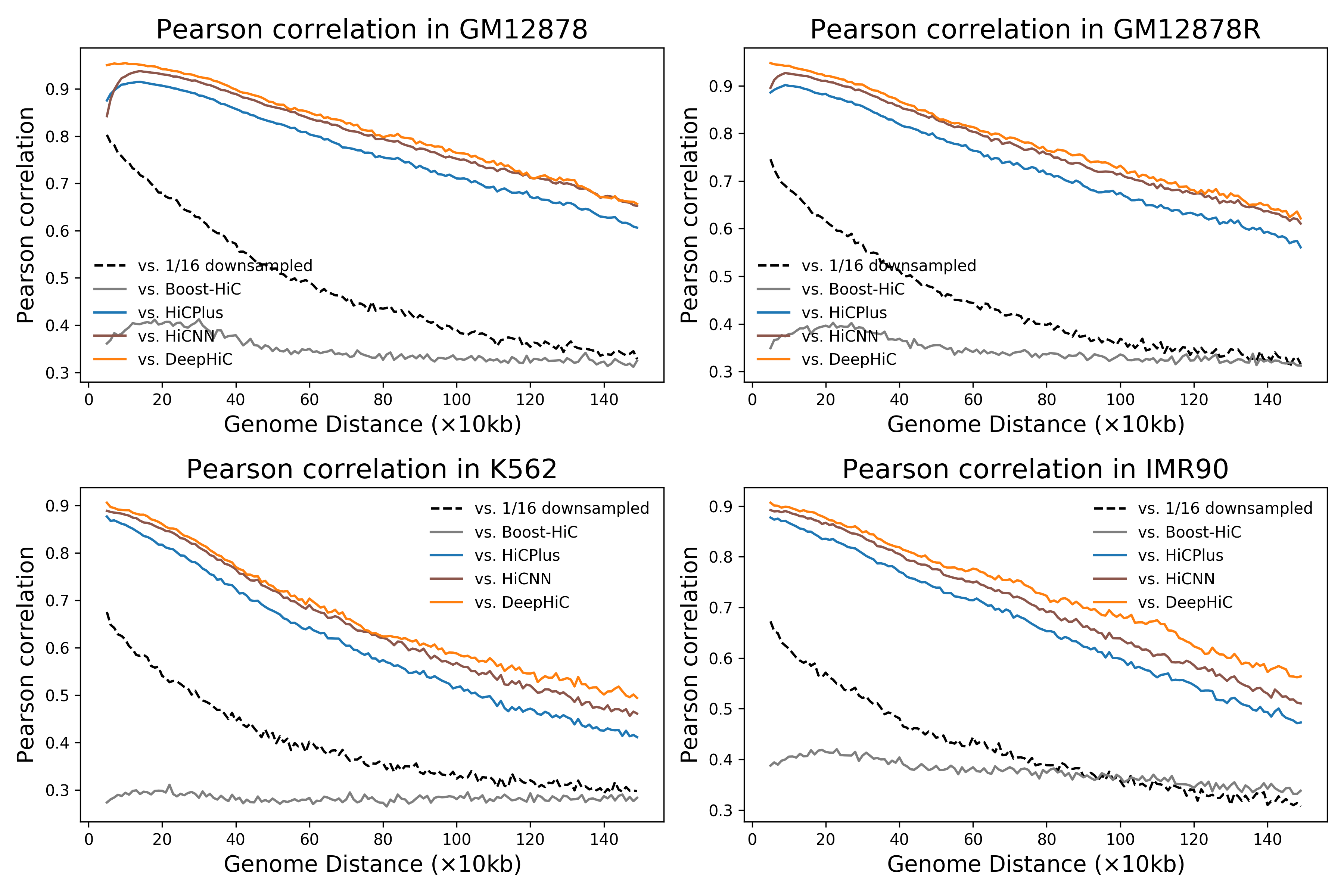

Supplement: S15 Fig — Between three types of non-experimental data: 1/16 downsampled, HiCPlus/Boost-HiC/HiCNN-enhanced, and DeepHiC-enhanced data in various cell types. We calculated the correlation three non-experimental data, as compare to real high-resolution data for all chromosomes at each genome distance. (TIFF) [file pcbi.1007287.s017.tiff]

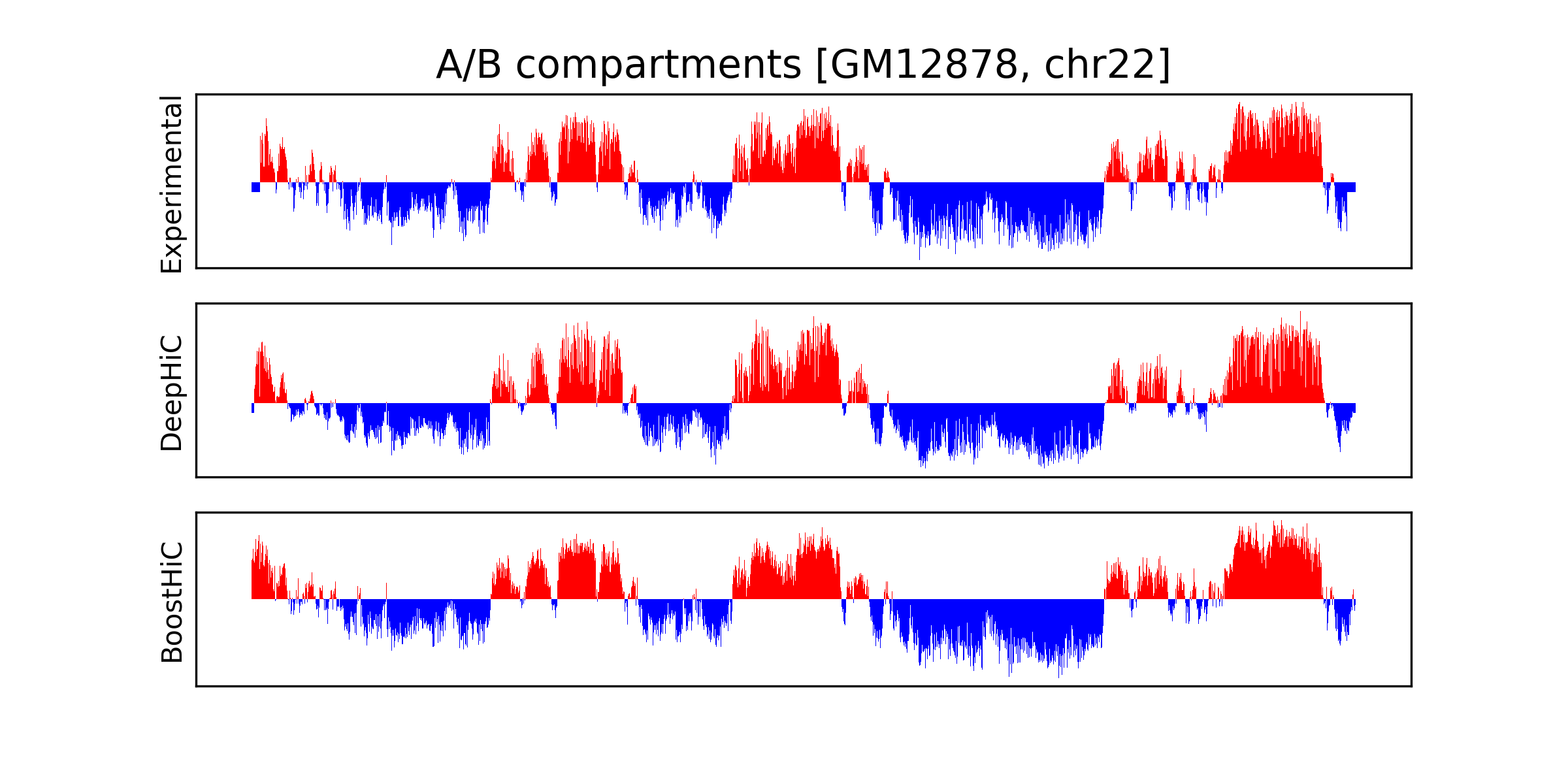

Supplement: S16 Fig — Quantitatively, correlation of DeepHiC and BoostHiC versus experimental are 0.952 and 0.966. And Jaccard indices are 0. (TIFF) [file pcbi.1007287.s018.tiff]

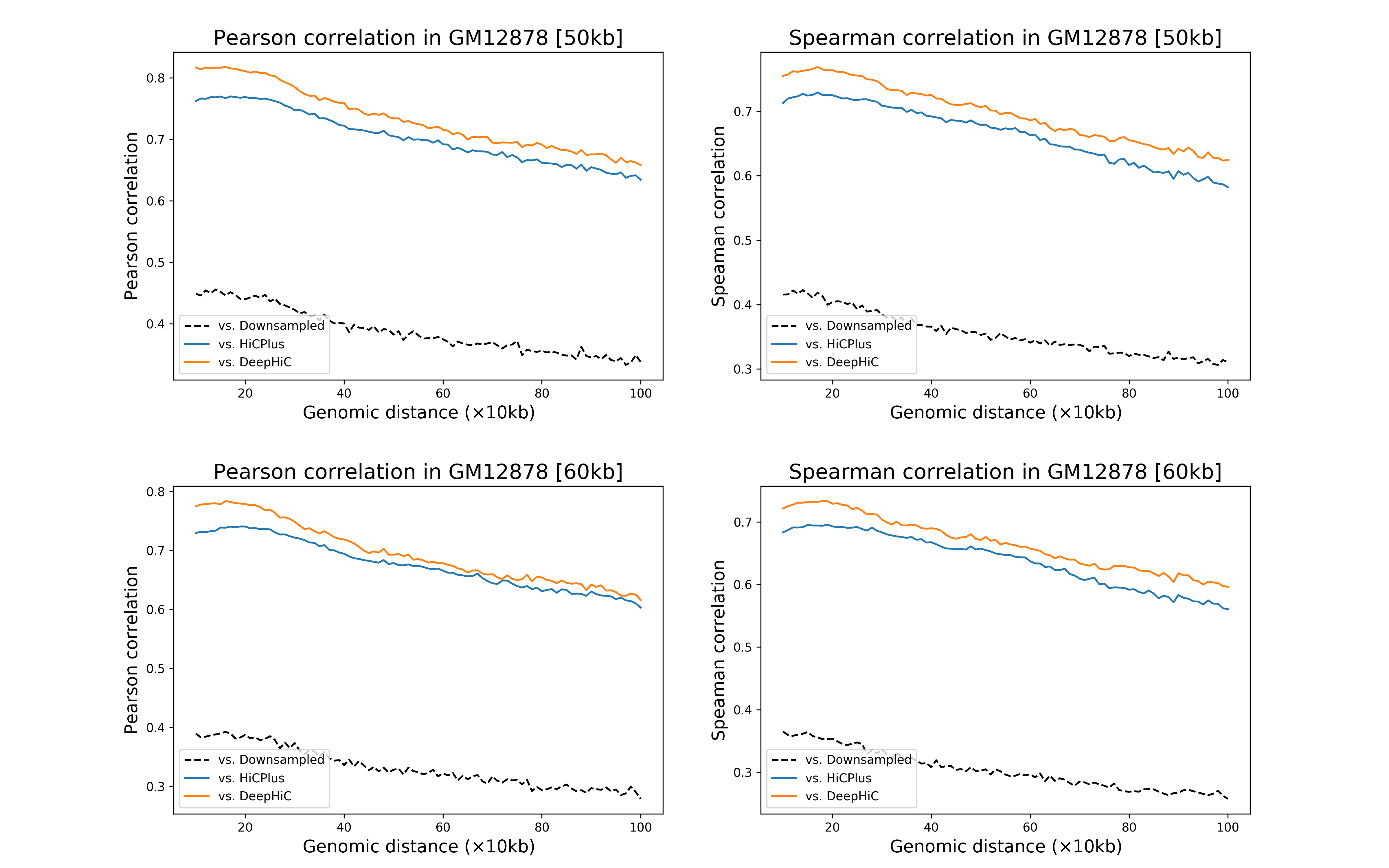

Supplement: S17 Fig — Correlations in GM12878 cell line when prediction 1/25 (50kb) and 1/36 (60kb) sequencing reads by using the trained model based on 1/16 sequencing reads. (TIFF) [file pcbi.1007287.s019.tiff]

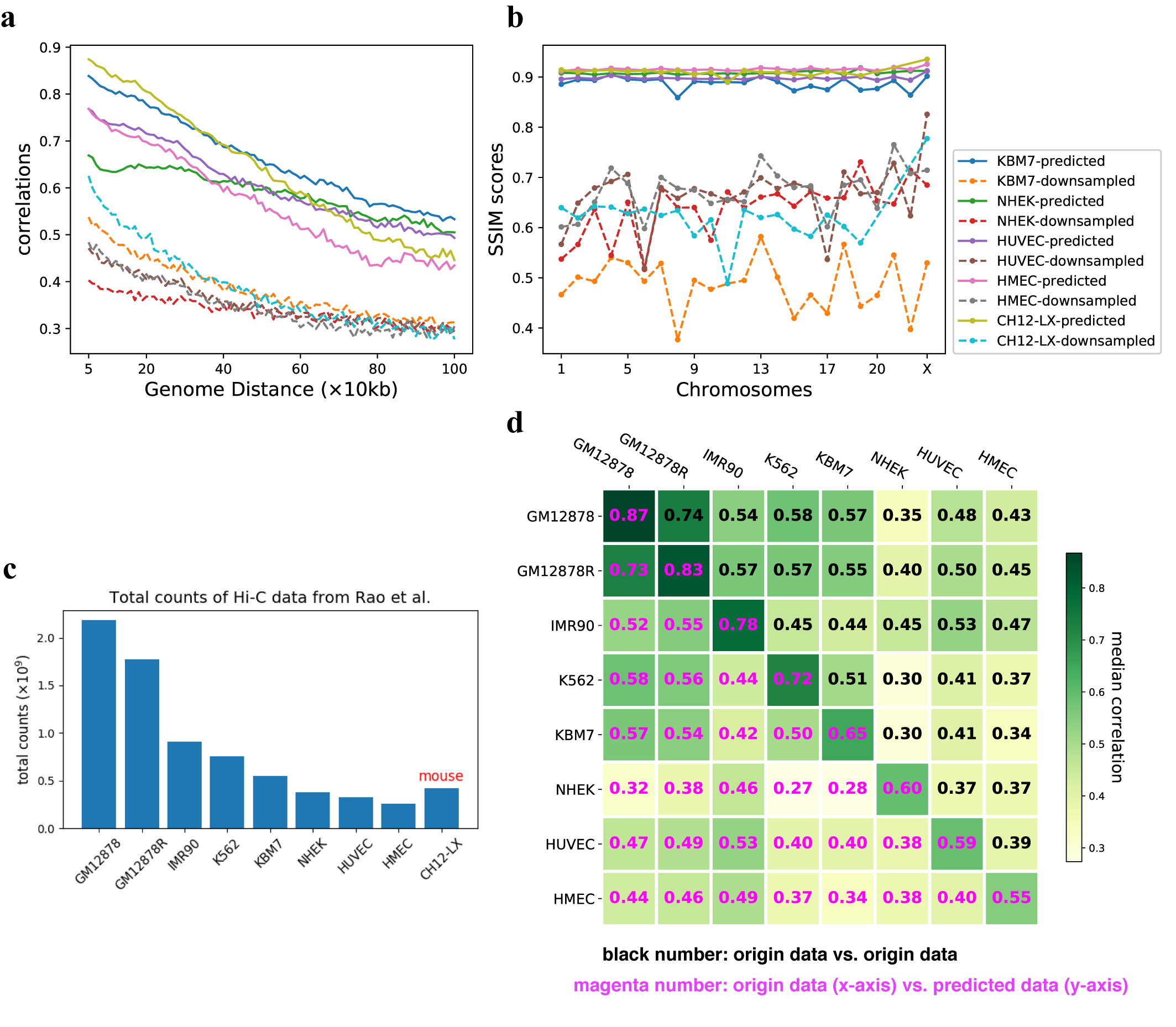

Supplement: S18 Fig — Performance of DeepHiC in other cell lines’ data from Rao et al., 2014 a, b) The performances of DeepHiC in KBM7, NHEK, HUVEC, HMEC, and CH12-LX cell lines, downsampling ratio is 1/16. c) Total read counts of 9 datasets (include 8 cell lines, the data of HeLa cell line is not available) from Rao et al., 2014. d) Correlations between Hi-C data across different cell types, black numbers represent correlations between two real data. Magenta numbers represent correlations between real data and predicted data (1/16 downsampled). Median correlation: median value of Pearson correlations from 50 kb to 1 Mb genomic distance (TIFF) [file pcbi.1007287.s020.tiff]

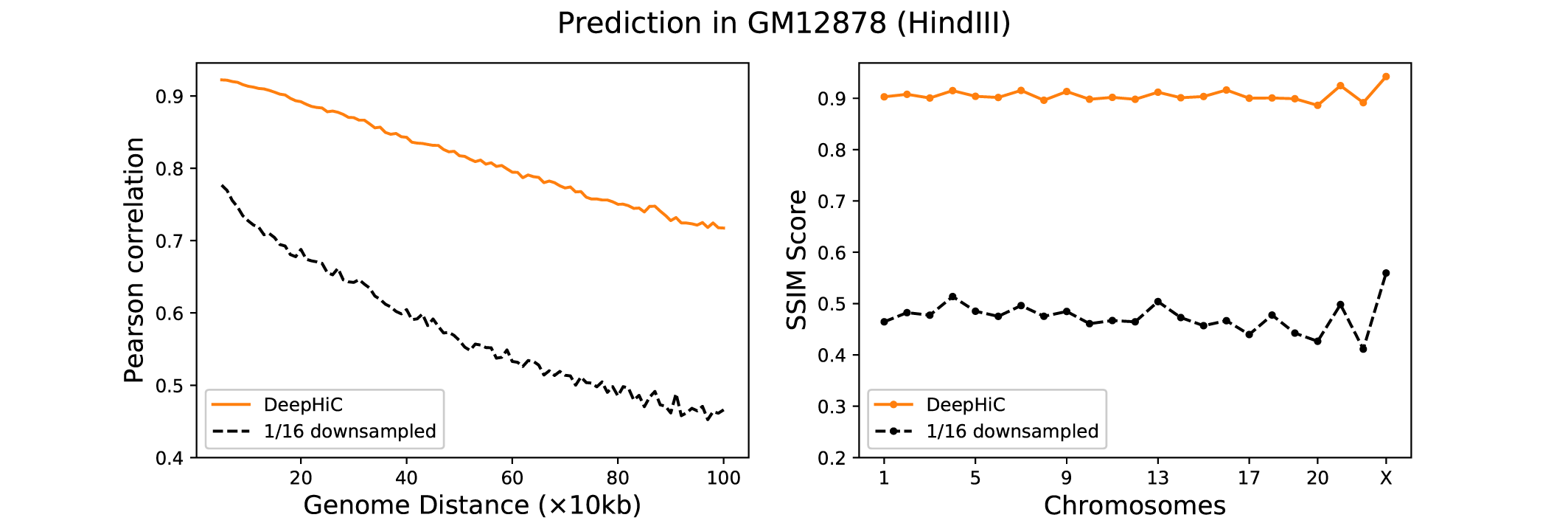

Supplement: S19 Fig — Performances of correlation and SSIM scores when apply DeepHiC to GM12878 HindIII(&Ncol) Hi-C data (dilution Hi-C, HindIII&NcoI, HIC034-037). (TIFF) [file pcbi.1007287.s021.tiff]

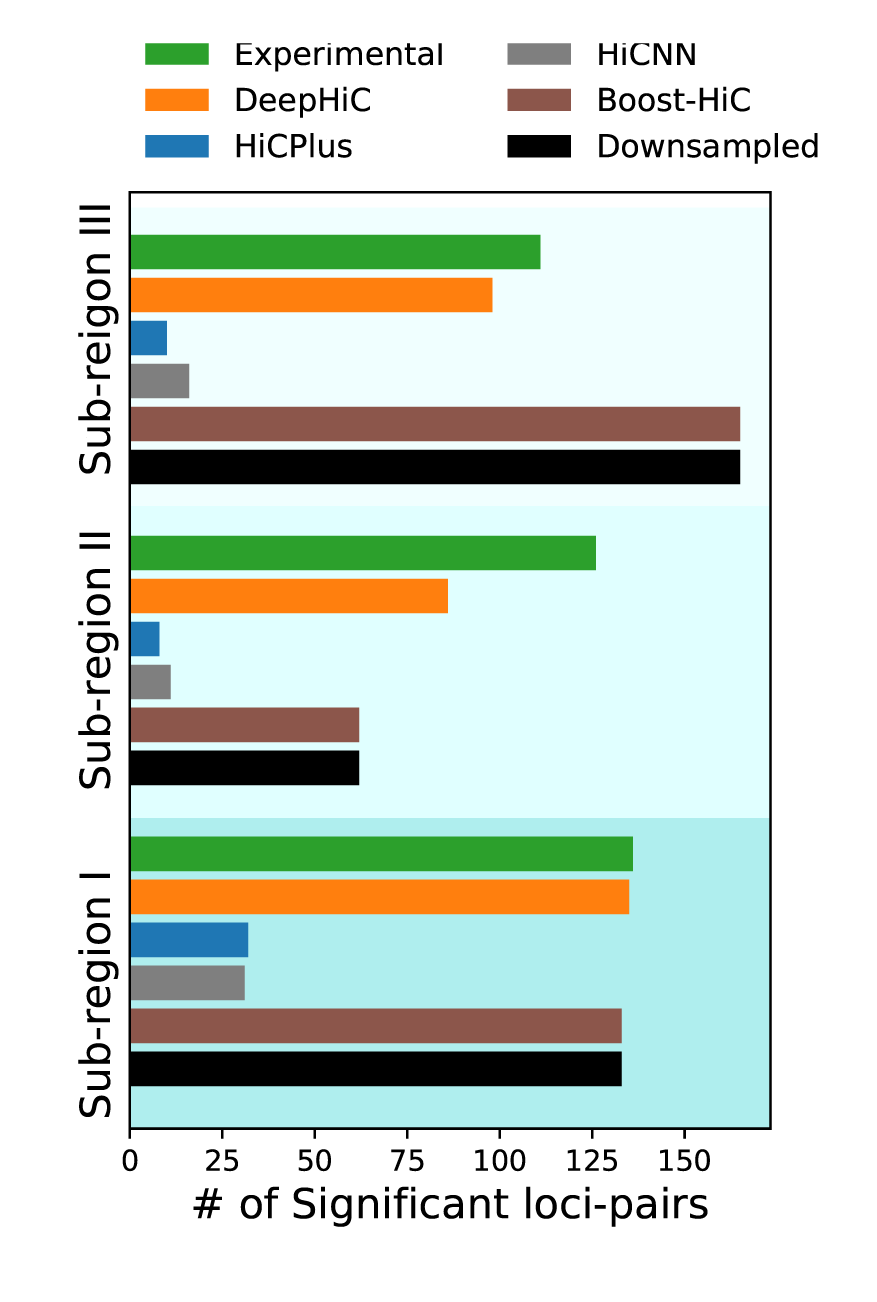

Supplement: S20 Fig — (TIFF) [file pcbi.1007287.s022.tiff]

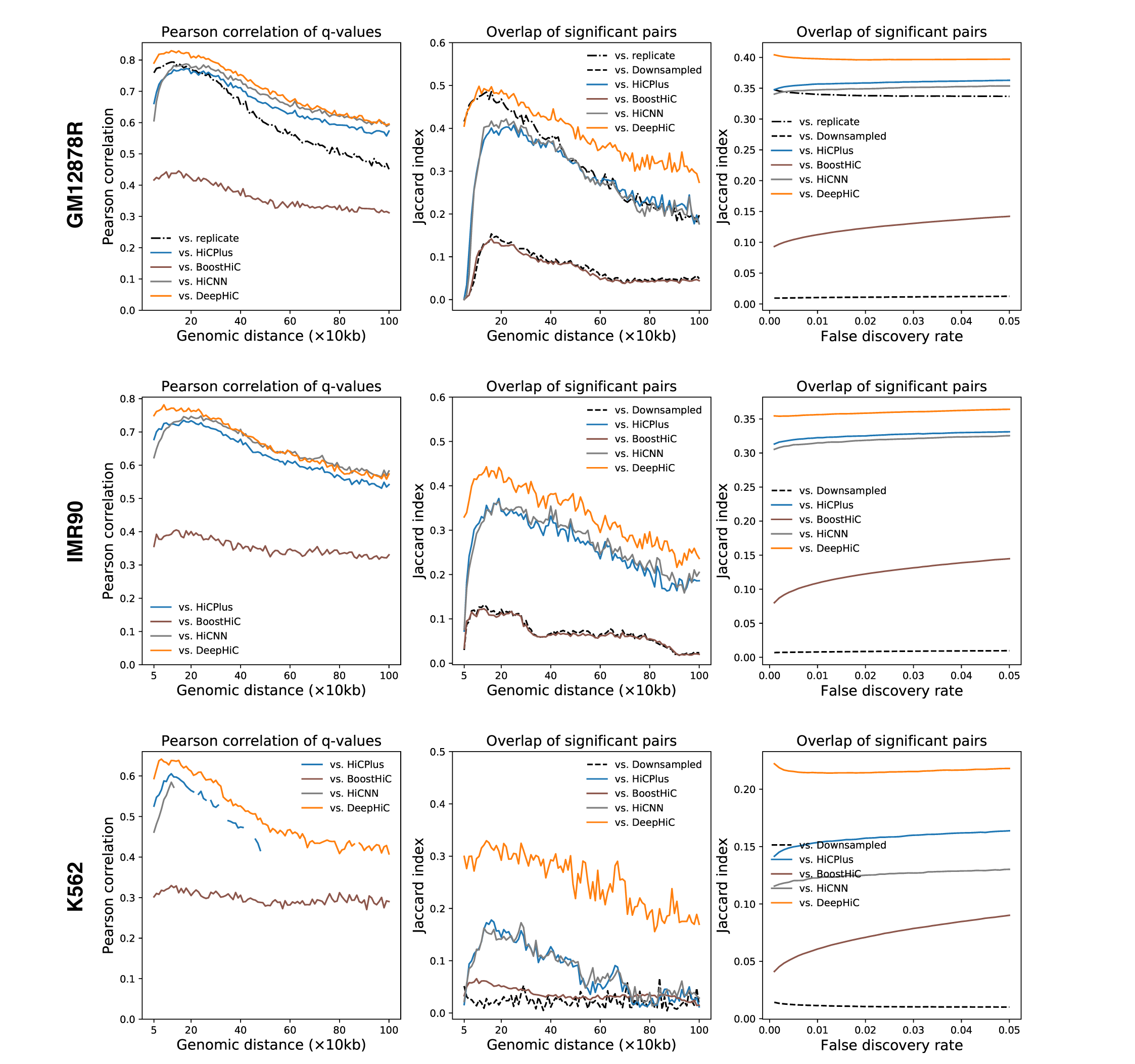

Supplement: S21 Fig — We investigated the correlation of significance matrices (first column), overlap of identified interactions with 0.5 percentile as cutoff (second column) at 100kb to 1Mb genomic distance in the GM12878R, K562, IMR90 datasets, together with overlap of identified interactions with different cutoff according to false discovery rate from 0.001 to 0.05 (third column) in those three datasets. (TIFF) [file pcbi.1007287.s023.tiff]

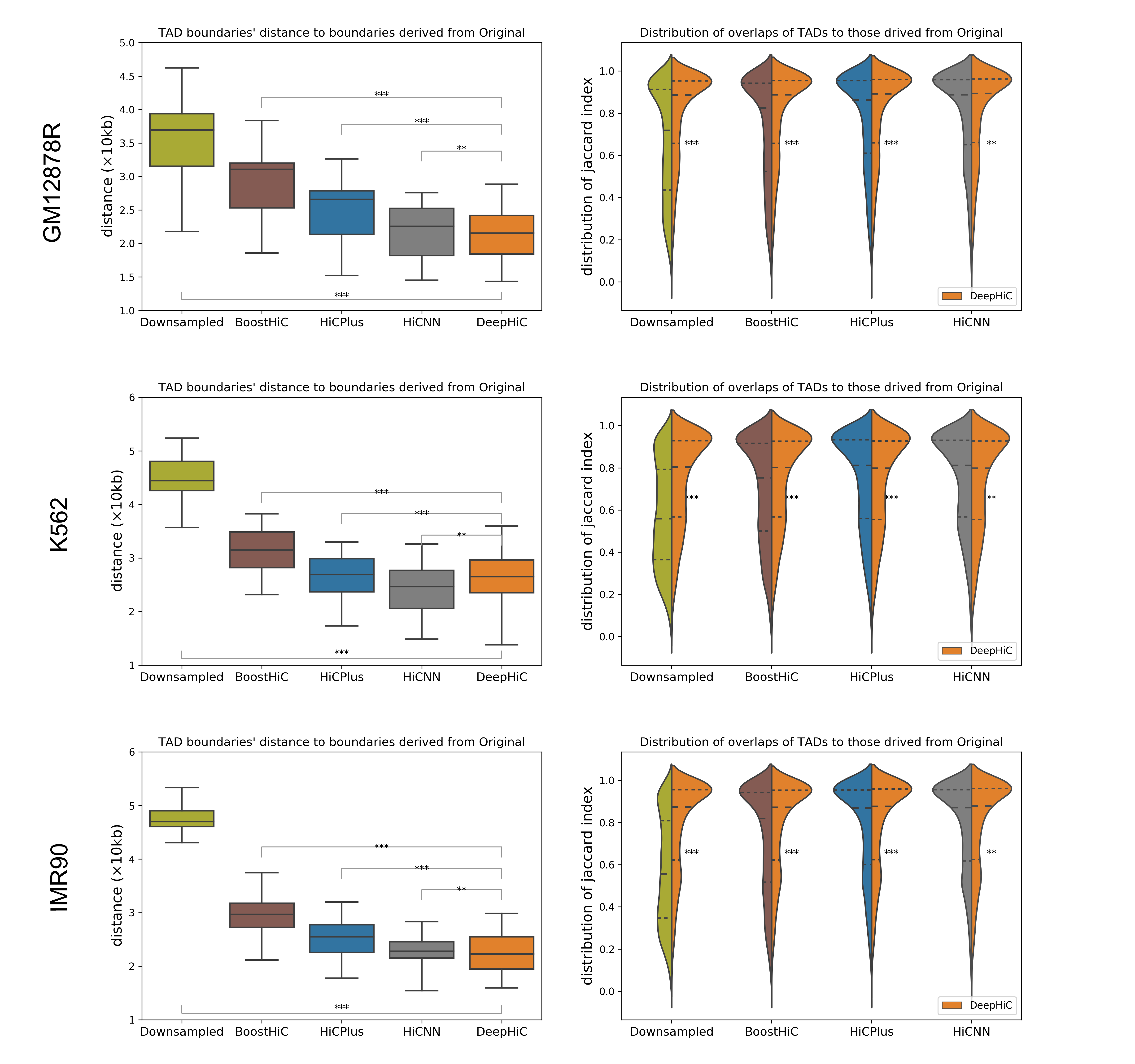

Supplement: S22 Fig — We investigated the performance in detecting TAD boundaries in GM12878R (first row), K562 (second row) and IMR90 (third row) datasets. ***: p-value < 1x10^-20^, **: p-value < 0.001, Mann Whitney U-test. (TIFF) [file pcbi.1007287.s024.tiff]

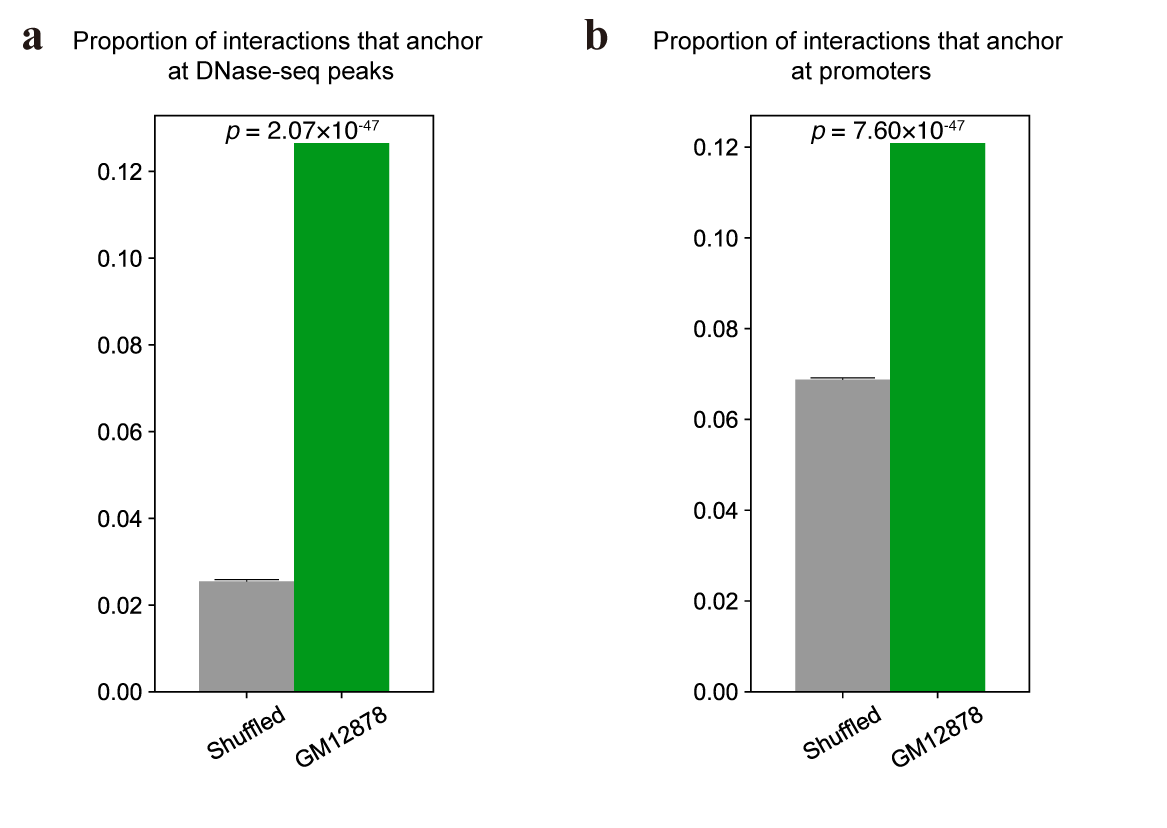

Supplement: S23 Fig — a) Fraction of significant interactions of which both connected loci contain DNase-seq signal peaks. Error bar: standard deviation. b) Fraction of significant interactions anchor loci which are interested with gene promoters. Error bar: standard deviation. (TIF) [file pcbi.1007287.s025.tif]

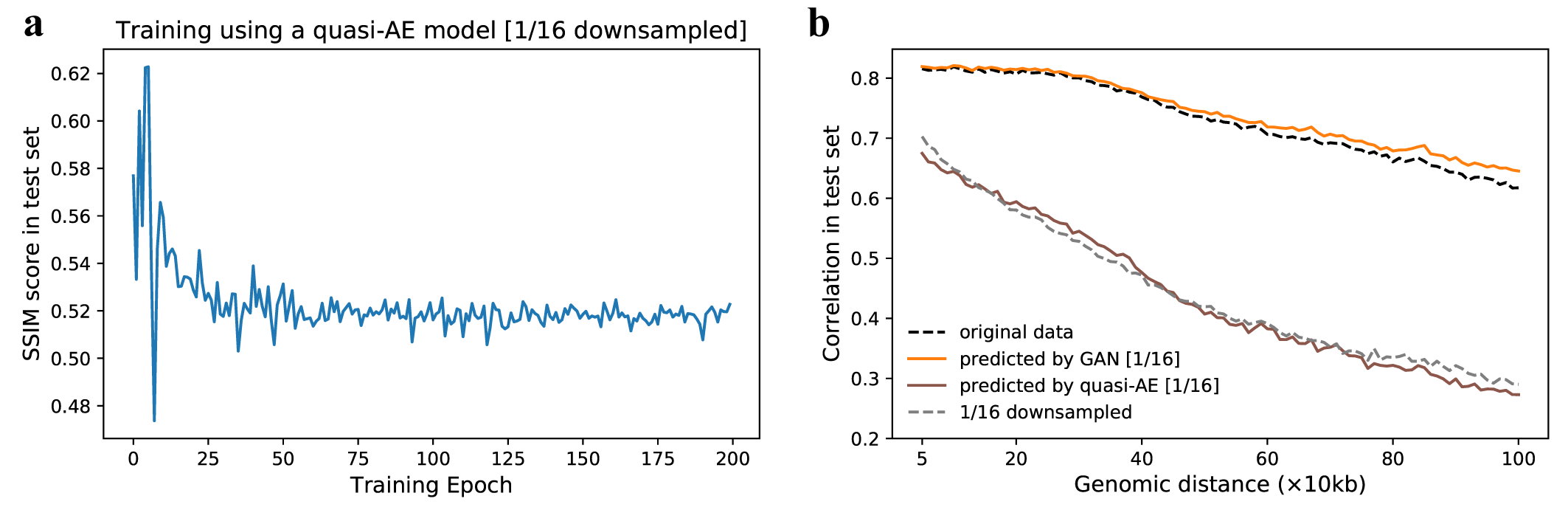

Supplement: S24 Fig — a) When training DeepHiC using a quasi-autoencoder scheme, the trends of SSIM score in test set. b) Using different trained manners, the performance of correlations between predicted-GM12878R versus original GM12878 dataset in various Hi-C data. (TIFF) [file pcbi.1007287.s026.tiff]
