# Supplementary material for: DeepHiC: A generative adversarial network for enhancing Hi-C data resolution
Source: PLoS Comput Biol. 2020 Feb 21;16(2):e1007287. doi: 10.1371/journal.pcbi.1007287 (PMC7055922; doi:10.1371/journal.pcbi.1007287)
Supplement: Attachment — Submitted filename: 2019DeepHiCReview.pdf [file pcbi.1007287.s029.pdf]

Review

Hong et al. present Deep-HiC, a computational approach to augment low coverage Hi-C data by predicting a high-resolution version using a generative adversarial neural network. DeepHiC integrates multiple loss functions to encourage both good reconstruction error and structural similarity (binary cross-entropy for the discriminator network, and MSE, perceptual loss and total variation loss for the generator network). The authors extensively benchmark the method, comparing it to state of the art models for the same task. They also show that the output of DeepHiC recapitulates known features of 3D genomics, such as TADs, compartments and significant interactions.

The paper is exceptional in its rigor both in terms of benchmarks and in terms of clarity of presentation. For example, separating training and test sets by chromosome ensures that there is no contamination between these to artificially inflate performance estimates. Figures extensively show the predictions and how they compare across methods. Performance benchmarks are summarized both globally but also stratified by distance between contacts or by type of genome 3D feature.

I consider this study to be an excellent match for PLoS Computational Biology, pending a few revisions as described below. My main concern is related to the fact that DeepHiC appears to achieve a similarity to the ground truth that is higher than that between experimental replicates, which is surprising since replicates which differ only due to sampling noise should be an upper bound for performance. My second concern, which is likely only related to the presentation of the paper, is to clarify whether a model trained in one cell type can be used (with good performance) in data from a different cell type or condition. I believe this is the case, but find that this distinction is not made clear in the text.

Finally, I suggest a few additional benchmarks to strengthen the paper, as well as some potential future directions for the authors to pursue outside the scope of this study.

Major comments

1. The key aspect that is not clear in the text is whether a model trained on data from cell type x can be used to predict in a new cell type, where only low resolution data are available. Making predictions extending beyond the cell type in which the model was trained is the key application for this approach. From the cover letter, it seems that indeed by training a model in GM12878 cells, they can make predictions for shallowly sequenced data from other cell types. Also, from the discussion, the same appears to be true: “DeepHiC may be used to approximate the real data, and to make predictions in other cell or tissue types.”. The authors should make it clear if this is the case in the manuscript, specifically by mentioning when they describe the performance of the model across cell types which cell type was used for training, and which for testing. This could be done in the section “DeepHiC reproduces high-resolution Hi-C from as few as 1% downsampled reads”.

2. A second key concern is why DeepHiC is able to perform better than a replicate experiment in GM12878. In theory, it should not be possible to perform better than a replicate experiment, as replicates differ from the original only in terms of sampling noise. It is imperative to understand why this is and report it in the text.
Below are a few hypotheses for how one could obtain better results with DeepHiC than from replicates.

- The sequencing depth of the two replicates is different. For instance, if replicate 1 used for training is sequenced deeper than replicate 2, then the model will predict with noise properties corresponding to the deeper sequencing depth, and the replicate experiment will have more limited power to detect the high resolution features that DeepHiC was able to learn from the deeply sequenced replicate. To address this, the authors can subsample the more deeply sequenced replicate to the sequencing depth of the other one, then train a model where the high-resolution data input is the subsampled replicate, and then compare with the other replicate.
- There is actually a difference in a property of the data between the two replicates, and because DeepHiC learns this property, it is able to predict it better than the replicate experiment can. One such property may be the distance dependence of contact frequency. The authors can check if the distance dependence curves are different between the two replicates, and if they are, re-sample one replicate using the distance dependence curve of the other, and then perform the performance comparison. Other properties may be the restriction enzyme used, exact protocol, etc. which are listed in Rao et al. 2014, supplementary information in Supplemental Table S1.
- There are clonal differences between the two replicates which have structural variation that accounts for the differences in HiC profiles. For example, Rao et al., 2014 describe the following in the supplement: “Note that, to make our data maximally comparable to that of the ENCODE project, all of our GM12878 cells were obtained from Coriell’s “Expansion A”, from a lot set aside for ENCODE. The cells derive from two different batches. One of the biological replicates in our dilution experiment (labeled HIC034, br10) and one other biological replicate (HIC048, br 13) derive from the first batch (“Batch 1”, received at the Broad Institute on 3/11/2008); the rest of our biological replicates derive from the second batch (“Batch 2”, received at the Broad Institute on 5/13/2008).”

3. Related to the above comment, how variable are the predictions as a function of restriction enzyme used?

4. There are a few additional benchmarks that would strengthen the paper, specifically showing the specificity of predictions across different conditions. A general concern for methods predicting HiC data is that if a model predicts an average HiC profile across cell types, it will look like a strong model since most TADs are constant between different cell types. Thus, it would be instructive to show that the model is able to pick up on cell type specific differences, as defined from the high-resolution data, for example changes in TAD boundary locations or

appearance/disappearance of TADs. Conceptually, DeepHiC should indeed correctly identify cell type specific features, since during training it only sees one cell type, and thus does not “know” how features such as TADs vary between cell types. However, it would be useful to confirm this analysis to the paper, as it would increase the confidence of the community in the method.

5. The authors show the performance across 4 cell types, even though Rao et al., 2014 have profiled 9 cell types. Please report the performance across all cell types in that study.

6. Conceptually, DeepHiC should generalize across species. Please show if this is the case.

7. The authors mention that they have implemented a parallelized version of Fit-Hi-C. Please confirm that the re-implementation and the original code produce the same results on a test dataset.

8. When comparing DeepHiC with Fit-Hi-C called interactions, I am not sure I understand the procedure. Fit-Hi-C requires the user to provide counts for each interaction. Please clarify how the output from the neural network is converted to counts, or alternatively what inputs are provided to Fit-Hi-C.

9. It is not clear how the authors set a cutoff for significant interactions called by Fit-Hi-C. The authors write “We kept the predicted significant interactions (q-value < 0.5-percentile) for genomic distances from 20 kb to 1 Mb for further comparative analysis.”. Does this mean that they take the top 0.5% of interactions, as sorted by q-value and call those significant? Or that they set a threshold on the q-value of 0.05? To my understanding, a concrete threshold should be set on the q-value, rather than selecting the top x% interactions. If we consider a dataset with reads uniformly distributed in the contact map, then there are no significant interactions, but the approach of selecting the top x percentile would produce a set of interactions. Please either modify the procedure to use a concrete q-value threshold, or clarify in the text if this is what was done.

10. It would be useful to translate the sequencing depth from fractions (e.g. 1/16) to the actual sequencing depth for the dataset, so that readers/users can assess the amount of sequencing required to use DeepHiC. Please specify in the discussion the lower bound of reads required for DeepHiC performance to be high.

11. In Fig S6, why are SSIM scores so bad across chromosomes for the replicates? And why are they so variable? I wonder if there is a technical artifact accounting for this.

12. The biological validation in Figure 6. The authors compute Fit-Hi-C significant interactions and measure the performance as the fraction of significantly called Fit-Hi-C interactions that anchor at i) promoters or ii) at ATAC-seq peaks. It is not clear to me that this is a valid biology-related performance metric. If DeepHiC were to connect all promoters and all ATAC-seq peaks, it would get a high score by this metric without being biologically sound. To alleviate my concern, the authors do check whether this assumption holds true in deeply sequenced HiC data, which is the case, compared to a shuffled control. In this case, the authors can suggest that the distribution of Fit-HiC calls is more similar to what would be obtained from a high-resolution dataset. While this makes the case that DeepHiC is more similar to deeply sequenced HiC data, it still in my mind does not say that the loops found using DeepHiC as an input are "better" biologically than those from the lowly sequenced data. Thus, the authors should clarify the wording when describing this section.
Related to this, in the introduction, the sentence " In this study, we applied DeepHiC to Hi-C data in mouse embryonic development and demonstrated that, compared with the original low-resolution Hi-C data, DeepHiC-enhanced Hi-C data provides more interpretable results for the identification for chromatin loops." should be revised. More interpretable does not necessarily mean correct.

13. A good validation for loops, as defined in Rao et al., 2014 would be to look at loops which are different across cell types. Since these loops are anchored by binding of CTCF, one would expect that when CTCF is absent in a cell type, the loop should be absent at a given site, and be present when CTCF is bound. By combining CTCF binding information and HiC the authors can check whether DeepHiC is able to correctly identify these differential loops.

14. The choice of applying DeepHiC to mouse early embryonic growth is very good, given that there is a limitation on the number of cells present, rather than just an economic motivation for low-resolution data.

15. For the ChIA-PET analysis in the section "Chromatin loops in high-resolution Hi-C were accurately recovered from DeepHiC-enhanced matrices", the authors describe their selection of the negative sets as follows: "As for negatives, we randomly selected the same number of loci pairs that were not predicted to be interacting pairs by ChIA-PET (10 repeats).". It would be more accurate to use a set of interaction pairs that are matched to the positive set in terms of distance between them.

16. "We mainly focused on the 8-cell stage and beyond because Hi-C data from earlier stages only demonstrate weak TADs and depleted distal chromatin interactions". Please show the analysis for these as well. If there are no TADs, then we would expect to see fewer interactions. Also, the structure is more different than what the model has seen in the training set, and thus it will be interesting to see the performance of DeepHiC.

17. What is the lowest size of genomic bin that can be used with DeepHiC? Does it work well at 5kb resolution? What about 1kb? Please add a figure showing performance as a function of resolution of the data at which the training is done.

18. The repository at https://github.com/omegahh/DeepHiC is very well written. It would further benefit from showing an example tutorial for running the model on a dataset that is not in the format of Rao et al., 2014. For instance, the authors could describe clearly the formats in which they expect the data to be, and describe in more detail what the different arguments are for the scripts being used.

19. The exciting performance of DeepHiC suggests it may do just as well predicting the rest of the contact matrix off the diagonal (i.e related to compartments). A future direction (out of the scope of the current study, perhaps) is to use the same model, or a separately trained model to predict the rest of the contact map, in order to obtain better resolution for the borders of A/B compartments, as well as for inter-chromosomal contacts.

20. It would be useful to check how distance dependence differences between datasets are preserved by DeepHiC. For instance, during the cell cycle, the scaling of contact frequency changes dramatically. It would be useful to confirm that DeepHiC preserves this difference.

21. Figure 3c shows that DeepHiC outperforms other methods when stratified by distance. It appears that there is a large gap in performance between DeepHiC and HiCNN at very small distances (<20kb), and that after 20kb, the performances between the two methods become much more similar. It would be useful to understand why DeepHiC performs better at low distances.

Minor comments

22. Be precise when using the word loops. “Loops” has been used to describe multiple different features of Hi-C maps. Specifically, some papers call chromatin contacts that significantly deviate from the expected contact frequency as loops, whereas other papers understand loops to be regions of enriched contact frequency relative to the surrounding chromatin (e.g. as defined in Rao et al., 2014).

For example, in the section “Application of DeepHiC improves identification of chromatin loops in mouse early embryonic developmental stages”, the word loops is used to mean both definitions: “Chromatin loops regulate spatial enhancer-promoter contacts and are relevant to domain formation [4, 53], and anchors of chromatin loops co-localize with open chromatin regions including insulators, enhancers, and promoters.”. Please revise to make the text more clear.

23. I would modify the name of DeepHiC to include a reference to the fact that the model is GAN. However, this is just a suggestion and I leave it to the authors to make the final decision.

24. The authors mention that “Because of the high cost of sequencing, most available Hi-C datasets have relatively low resolution, which limits their application in studies of genomic regulatory elements”. Define low resolution in the text and give a number to demonstrate the limited applicability for studying regulatory elements.

25. “Low resolution data may be less effective for detecting large-scale genomic patterns such as A/B compartments”. Compartments are frequently called at 1Mb resolution, so I would say that compared to other 3D genome features, this is actually effectively estimated even with low coverage.

26. I can only see the figures in low resolution, which makes it difficult to properly visually assess Figures 2, 4.

27. Please clarify the normalization scheme needed for the inputs to DeepHiC (sqrtvc, KR, etc.). Ideally DeepHiC is applied to normalized data, rather than raw data that is biased by uneven coverage of genomic bins.

28. In Figure S17, add replicate as a another benchmark.

29. The authors refer to downsampled data as low-resolution data. While it is true that having fewer reads warrants using a lower resolution, the authors use the low-resolution data binned at a high resolution which can be confusing to the reader. To avoid this confusion, I would suggest to use “low coverage” (which the authors use sometimes) rather than “low resolution”.

30. For Boost-HiC, did other parameters of alpha result in better performance?

31. The sentence “DeepHiC can be used to enhance the resolution of existing time-resolved Hi-C data obtained through early embryonic growth. These data are prone to low resolution due to limited cell population.” should include in parentheses the resolution that was used.

32. The A/B compartment analysis described in “DeepHiC outperformed other methods in terms of genome-wide similarity”” would be strengthened if it were summarized with a statistic related to figure S14, for instance the percent of genomic regions assigned to the same compartment between two methods, or the correlation between compartment scores.

Also, Figure S14 is incorrectly referenced as Figure S4 in the relevant paragraph - please correct this.

33. Please add a sentence in the methods section describing how the ChIA-PET data were processed, in addition to the currently present description of where they were downloaded from. How were significant interactions defined? And how were these “assigned” to Hi-C bins in later analyses?

34. I recommend a careful read through the paper to correct any typos or other small errors such as incomplete sentences.

35. For Figure 2, please add numbers of the colorbars to denote the upper and lower bounds. Currently, these are labelled as “high” and “low”.

Future directions outside the scope of the current study

A direction for future work would be to apply such a model to very high resolution 3D genome data such as MicroC-XL.

Another future direction will be to check the applicability of DeepHiC to single cell HiC data.

Finally, since DeepHiC’s impact is the largest in cases where it is challenging to collect deeply sequenced HiC data, it would be interesting to check how DeepHiC enhances allele-specific contacts (e.g. as described and computed in Rao et al., 2014). Allele-specific contact maps rely on SNPs to annotate the allele for the reads in the dataset, resulting in severely diminished numbers of reads ultimately being used.
