# Supplementary material for: DeepHiC: A generative adversarial network for enhancing Hi-C data resolution
Source: PLoS Comput Biol. 2020 Feb 21;16(2):e1007287. doi: 10.1371/journal.pcbi.1007287 (PMC7055922; doi:10.1371/journal.pcbi.1007287)
Supplement: Attachment — Submitted filename: Response_to_Reviewers.docx [file pcbi.1007287.s030.docx]

Dear editor,

We appreciate the handling of our manuscript titled “DeepHiC: A Generative Adversarial Network for Enhancing Hi-C Data Resolution” and the opportunity given to us to prepare a revision. All the comments of referees are very helpful, and have been incorporated into the revised manuscript. The manuscript has been improved according to the referees’ comprehensive remarks. Besides the reviewers’ useful comments, we have examined our manuscript carefully, and have revised some improper expressions.

We thank the referees for their helpful comments and hope that the revised manuscript is acceptable for publication.

Thank you very much for your help. I will be glad to supply any additional information that can be of assistance.

Sincerely yours,

Xiaochen Bo

**The response is organized as follows:**

- **Response to reviewer #1**
- **Response to reviewer #2**
- **Response to reviewer #3**

(Figures in this response letter are indexed using Supp Fig. X)

**Response to reviewer #1**

*Comments to the Authors:*

The authors present a method, named DeepHiC, that aims to increase the effective sequencing depth of Hi-C experiments. The method relies on a pair of neural networks that are trained in an adversarial manner similar to image super-resolution. While the paper is very clearly written and easy to follow, there are several critical issues that should be addressed. Further, while the authors demonstrate in several contexts that DeepHiCs outperforms previous work, the manuscript may benefit from a further discussion of the ramifications of these improvements.

**Response:** We thank the reviewer for the thoughtful comments. All suggestions have been incorporated into the revised manuscript. We have improved our manuscript according to the helpful comments, some very important experiments have been added, the definitions and descriptions in the manuscript are more accurate and more detailed. Our response to the comments is as follows:

**Major comments**

- While the general problem of increasing the effective sequencing depth of Hi-C experiments is important, the specific formulation considered by the authors is problematic. Specifically, it will never be the case that one has high resolution data for a few chromosomes and needs to improve the quality of the remainder (the cross-chromosomal setting). Rather, a user would like a model that had been trained on high quality data from one cell type and could then be used to improve the quality of other cell types (the cross-cell type setting). This distinction is important because, in the cross-cell type setting, a strong baseline is simply using the high-resolution contact map from the cell type used to train the model. The Hi-C+ paper addresses this setting explicitly.

**Response**: We thank the reviewer for pointing this out. In the revised manuscript, we added a paragraph to describe how we performed training and testing at the end of the second result section. In brief, we only trained DeepHiC on the data in GM12878 cell line (refer to ‘primary’ in Rao’s Supplemental Table S1, HIC001-018).

In particular, the model was trained on chromosomes 1-14 (training set) and tested on the remaining chromosomes in GM12878 cell line during the training process (we call it testing set instead of validation set because we didn’t perform model selection or hyperparameter tuning by using this dataset). Further applications of DeepHiC to other cell types and mouse embryonic data were still based on the previously trained model using GM12878. Besides, we also trained DeepHiC on different chromosomes’ data in GM12878 dataset (in S5 Fig) only to show that our model is independent to the chromosomes we choose for training.

Besides, DeepHiC can also be trained on *IMR90* and *K562* datasets, which are other two deeply sequenced data from Rao et al., 2014. As shown in Supp Fig. 1a, 1b. We can see that they both converge and achieve nearly the same correlation as using GM12878 for training data. Noting that the *GM12878* dataset is the deepest sequenced Hi-C data (Supp Fig. 1c).

**Supp Fig. 1**

**Supp Fig. 1 Legend:** **a**) The SSIM scores in test set (chr15-chrX) are approaching nearly 0.9 when training DeepHiC on chr1-chr14 data in the K562 or IMR90 cell line. **b**) Pearson correlation in the same set (chr15-chrX in IMR90) when using trained model from different cell type. **c**) Total read counts of 9 datasets (include 8 cell lines, the data of HeLa cell line is not available) from Rao et al., 2014.

- The authors should clarify what precisely constituted the ground truth labels. Specifically, was the Hi-C data that had outliers removed and then min-max scaled used solely for training, or was it also considered to be the ground truth? Considering these transformed values to be the ground truth would be unfair to the competing models that had been pre-trained on data sets which were processed in a different manner. Supplementary Note 1 suggests that the output from the models are not converted back to the original space because their ranges differ, with DeepHiC ranging from 0-1, and other methods ranging from 0-100. Further, the output from the webserver is between 0 and 1. It is okay for DeepHiC to be trained using data processed in a different manner than other approaches, but all approaches should be evaluated on the original Hi-C counts to ensure fairness. Additionally, because most outliers tend to occur on at low genomic distances, squashing these outliers only for DeepHiC may help explain why it outperforms the other models by so much at low genomic distances.

**Response:** Thank you very much for the comments. First, the high-resolution ground truth is the original Hi-C data in evaluation. The outliers in high-resolution data were removed only in training. We have revised the manuscript to make it more clear.

Second, HiCPlus and HiCNN also performed squashing to handle the outliers according to their codes. Especially, for the low-coverage input matrices (1/16 downsampled), we used the same cutoff value 100 as these methods, except that we performed the max-min normalization.

Third, we keep the DeepHiC output ranging from 0-1 unless it is required to be converted to integer. For example, like Fit-Hi-C, integral counts are required for input. We can convert them to 0-255 by multiplying 255. It makes sense that DeepHiC is trained to fit the contact map ranges from 0-255 before min-max normalization. And it can be easily verified by comparing the sum value between high-resolution contact map and predicted contact map in other datasets. For example, after max-min normalization, the sum value of the former is 2176479.15, and the latter is 2259598.32, which is 1.03 times to the former, in *K562* dataset. We have added this detail to the revised manuscript.

- It is unclear that performing better than an experimental replicate by such a large margin across all genomic distances is a reasonable result. The authors should comment on what such a result means and why there is such large variance between replicates. Generally, one would think of an experimental replicate as almost an upper bound of performance. This result suggests that it is significantly better to use DeepHiC on a Hi-C map with low sequencing depth than to perform the actual high sequencing depth experiment. This may be the actual result, but it is a significant claim and should have more supporting evidence.

**Response:** Thank you for these comments. At first, please allow us use a diagram to show four different schemes of calculating correlations between replicates, as shown in Supp Fig. 2a, for addressing your concerns. Noting that the replicated datasets we used are the ‘primary’ (HIC001-018), refers to GM12878, and the ‘replicate’ (HIC019-029), refers to GM12878R, from Rao et al., 2014. Their correlation in test set (chr15-chr22) are black dash lines in Supp Fig. 2b-e. The sampling noise in experiments may cause the difference between replicates.

1. The Supp Fig. 2b was the figure we used in the manuscript. It showed the correlations between enhanced-Hi-C from various downsampling ratios versus the original Hi-C in test set. These results revealed the ability of our model in fitting to the ground truth in terms of generalization. Together with the same results in K562 and IMR90 cell lines, it revealed that our model could fit to the ground truth data when using its downsampled inputs. Thus, the black dash line in Supp Fig. 2b has no relationship with colored lines. Imaging that there is a worse/better replicate data, performance of fitting to the ground truth should not be influenced by the replicate. In conclusion, the black dash line in this doesn’t make sense, colored lines indicated that our model’s performance decayed when fitting to the ground truth along with the downsampling ratio increased.

2. The Supp Fig. 2c-d are the circumstances that happen in reality. For instance, we had a deeply sequenced data GM12878, then we sequenced a replicate in low sequencing depth, which could be downsampled-GM12878R. By DeepHiC imputation, the correlation of enhanced data is as good as the real replicate, which is shown in Supp Fig. 2d. Or in Supp Fig. 2c for the opposite. Further, if we have two low sequenced Hi-C data, enhancement on both of them could also improve the correlation between them, as shown in Supp Fig. 2e. The slight superiorities may suggest that our model is capable of denoising, which is also claimed in Goodfellow et al., 2014 (the original GAN paper). In the revised manuscript, we replaced Figure 1d with Supp Fig. 2c.

**Supp Fig. 2**

**Supp Fig. 2 Legend: a)** diagram of four ways to calculated correlations between replicates shown in **b-e**. **b)** is the Figure 1d in old manuscript. **c-e)** correlations between ‘primary’ and ‘replicate’ data in predicted/original/downsampled datasets in the test set (chr15-chr22) in different ways according to the diagram in **a**.

- When a researcher has a Hi-C map with moderate coverage, they will likely want to know by what factor they should improve the effective coverage of that map in order to discover various features of chromatin architecture. For example, should they use a model that improves coverage by 100x (by training using 1% of the reads) or only by 16x (by training using ~6% of the reads)? Is it always better to use a model that improves coverage the most, or does improvement plateau after a certain amount of boost? It would be useful to readers if the authors could provide guidance on which model is appropriate in which setting, preferably by showing the performance on downstream tasks of models trained to boost coverage by different amounts.

**Response:** Thank you for your suggestion. Translating the downsampling factors to read coverage or data distribution is indispensable for researchers/users. We add a supplementary note for this in the revised manuscript. Considering that there is no concrete criterion of 10-kb Hi-C data, the strategy of choosing model should be related to the training data we used. The detailed answer is listed in the following:

1. **Users could choose the model according to the distribution of their Hi-C matrix**, especially focus on the values of top 95-99.9 percentiles (in all non-zero values). As shown in Supp Fig. 3a, for different downsampled data, the cutoff values for low-coverage input (*-lrc* parameter for our code in GitHub) were close to the 99.9 percentile values in our trained data. For a low-coverage data, user should use the model where their 99.9-percentile value is close to or lower than the cutoff value of chosen model. A caveat is that user should not use 25 as a cutoff value while the 99.9 percentile of their data is 100. Therefore, the model for 1/100 downsampled data is not always the best choice.

2. **If user has a Hi-C data which is latently in 1/16 to GM12878 data, it should be better to use the model trained on 1/16 downsampled data.** We downsampled GM12878 data to 1/25, 1/36 ratio, but still using the model trained in 1/16 downsampled data. These results have been shown in Fig. S17. Our model still worked well but performance decayed.

3. **Another important metric is the non-sparsity of input data must greater than ~10%.** Since our low-coverage input is still binned at 10-kb, the non-sparsity (#non-zeros/#all-pairs) of input decreased when the downsampled factor increased, as shown in Supp Fig. 3b. That means for 1/100 downsampled data (non-sparsity=9%), our model imputes ~75% cells from zeros to fit the original data (non-sparsity=84%). A lower non-sparsity of input data reaches the lower bound of our current model. Thus, prediction in 1/100 downsampled data of K562 and IMR90 didn’t worked because their 1/100 downsampled data are in lower non-sparsity (Supp Fig. 3c)

**Supp Fig. 3**

**Supp Fig. 3 Legend: a)** The trend of top 95-99.9 percentiles of non-zero values in GM12878 downsampled data. The cutoff value is used for model input. **b)** The non-sparsity of training data in different downsampling factors. **c)** The non-sparsity of IMR90 and K562 data in different downsampling factors.

**Minor Comments**

- The webserver did not work when tried

**Response**: Thank you for point this out. We have checked and tested our webserver many times and it works fine now. We also asked some friends in different countries to test the accessibility and ran the demo several times. Moreover, we have refined the guides to be more clear and friendly. Finally, a warning is that a large .npz file may be failed while uploading.

- An important distinction that should be investigated is whether the contact maps yielded by DeepHiC are better because they are effectively higher sequencing depth or because the generative network simply cleans the data. This question could be answered by training a model in the same manner as DeepHiC, but having the target be the same as the input, similar to an autoencoder. This model would demonstrate the performance one gets from cleaning the data. Thus, any improvement that DeepHiC has over it could reasonably be attributed to the importance of a higher sequencing depth.

**Response:** Thank you for the comment. We performed the test you recommended, as shown in Supp Fig. 4 (The autoencoder-like training scheme was named quasi-AE). The performance we get from the cleaned data is not as good as that of our model. It suggests that our model may truly improving the sequencing depth. We added this result in the discussion.

**Supp Fig. 4.**

**Supp Fig. 4 Legend: a)** When training using a quasi-autoencoder scheme, the trends of SSIM score in test set. **b)** Basing on the trained model, the performance of correlations between predicted-GM12878R versus original GM12878 dataset in various Hi-C data.

- The authors clearly describe the architecture, hyperparameters, and composition of loss functions of the models that compose DeepHiC. However, this naturally leads to the question of how such were determined. While it is not necessary for the authors to perform grid-search using a validation set, they should at least comment on how such were determined and clarify that they were not determined based on performance on the test set.

**Response:** Thank you for the comment. The network architecture and hyperparameters is not determined based on performance on the test set. At first, we must thank Ledig et al., for their enlightening work SRGAN, as we cited in the Introduction. The GAN framework and using perception loss is inspired from them. Secondly, there are not such many hyperparameters needed to be determined. We chose the convolution filter sizes, channels which are commonly used in many Deep Learning settings, like out-channels for convolutional layers are 64-128-256 is broadly used, like the VGG network. Other parameters like stride=1, it is the default manner in the most convolution layers, and padding=1 or 4 is result from what filter size and stride are. The hyperparameters in loss function were set to ensure each loss term on the same scale. There may be a better setting of hyperparameters that may lead to a better performance.

- The SSIM score is difficult to interpret as the primary evaluation metric. While the authors soundly reason that using MSE as the objective function during training may yield overly smooth predictions, there is no reason not to use it to evaluate trained models, particularly because it is much more interpretable to most readers.

**Response**: Thank you for the suggestion. Actually, we have calculated another metric related to MSE during training. It is peak signal-to-noise ratio (PSNR), which could be derived from MSE. Given two images X and Y of the same size, PSNR is defined as:

$$PSNR\left( X,Y \right)=10\cdot log_{10} (\left( 2^{n}-1 \right)^{2}/MSE(X,Y)),$$

in dB. Here, n is the number of bits for pixels in the image, which is equivalent to 1 in our experiments. As shown in Supp Fig. 5, the PSNR score reaches the plateau in the earlier epochs. They are about 35 dB, which means MSE is about 1e-3.5. We add this result to the revised manuscript.

**Supp Fig. 5**

- It is unclear how predictions were made in a genome-wide fashion. Was a cross-validation approach used, as alluded to in the methods? This should be explicitly stated in the text and include the number of folds. Similarly, in the final results section, the data set used to train the DeepHiC model should be briefly described.

**Response**: Thank you for the comment. We revised the manuscript to make these more clear. First, predictions were made in genome wide to contact matrices where genomic distance < 2 Mb using the trained model. Second, we performed a 5-fold cross validation to check that training is consistent to training chromosome we used. Third, we applied the trained model on 1/16 downsampled data to mouse embryonic Hi-C in the last result section.

- The term "(q-value < 0.5-percentile)" is difficult to parse. Was it a q-value < 0.5 that was used, or were the bottom 0.5% of q-values used?

**Response**: Thank you for pointing this out. It was the bottom 0.5% of q-values used. Through bottom 0.5% of q-values are all far smaller than 0.001, we revised it to a concrete threshold. It is 1e-6, also used in HiCPlus paper. This change involves the Figure 4a, 4c and Fig S21, as well as Figure 6b, 6c, and 6d in last result section. We have re-implemented the evaluations and revised the manuscripts.

**Response to reviewer #2**

*Comments to the Authors:*

Hong et al. present Deep-HiC, a computational approach to augment low coverage Hi-C data by predicting a high-resolution version using a generative adversarial neural network. DeepHiC integrates multiple loss functions to encourage both good reconstruction error and structural similarity (binary cross-entropy for the discriminator network, and MSE, perceptual loss and total variation loss for the generator network). The authors extensively benchmark the method, comparing it to state of the art models for the same task. They also show that the output of DeepHiC recapitulates known features of 3D genomics, such as TADs, compartments and significant interactions.

The paper is exceptional in its rigor both in terms of benchmarks and in terms of clarity of presentation. For example, separating training and test sets by chromosome ensures that there is no contamination between these to artificially inflate performance estimates. Figures extensively show the predictions and how they compare across methods. Performance benchmarks are summarized both globally but also stratified by distance between contacts or by type of genome 3D feature.

I consider this study to be an excellent match for PLoS Computational Biology, pending a few revisions as described below. My main concern is related to the fact that DeepHiC appears to achieve a similarity to the ground truth that is higher than that between experimental replicates, which is surprising since replicates which differ only due to sampling noise should be an upper bound for performance. My second concern, which is likely only related to the presentation of the paper, is to clarify whether a model trained in one cell type can be used (with good performance) in data from a different cell type or condition. I believe this is the case, but find that this distinction is not made clear in the text.

Finally, I suggest a few additional benchmarks to strengthen the paper, as well as some potential future directions for the authors to pursue outside the scope of this study.

**Response**: Thank you very much for your nice summary of the manuscript, and for your helpful and professional comments to further improvements. According to the helpful comments, we have greatly improved the manuscript, and many analyses have been added. With the revised manuscript, we have responded point-by-point to all the concerns of the reviewers.

**Major comments:**

1. The key aspect that is not clear in the text is whether a model trained on data from cell type x can be used to predict in a new cell type, where only low-resolution data are available. Making predictions extending beyond the cell type in which the model was trained is the key application for this approach. From the cover letter, it seems that indeed by training a model in GM12878 cells, they can make predictions for shallowly sequenced data from other cell types. Also, from the discussion, the same appears to be true: “DeepHiC may be used to approximate the real data, and to make predictions in other cell or tissue types.”. The authors should make it clear if this is the case in the manuscript, specifically by mentioning when they describe the performance of the model across cell types which cell type was used for training, and which for testing. This could be done in the section “DeepHiC reproduces high-resolution Hi-C from as few as 1% downsampled reads”.

**Response**: Thank you for the comment. In the revised manuscript, we added a paragraph to describe how we performed training and testing at the end of the second result section. In brief, we only trained DeepHiC on the data in GM12878 cell line (refer to ‘primary’ in Rao’s Supplemental Table S1, HIC001-018).

In particular, the model was trained on chromosomes 1-14 (training set) and tested on the remaining chromosomes in GM12878 cell line during the training process (we call it testing set instead of validation set because we didn’t perform model selection or hyperparameter tuning by using this dataset). Further applications of DeepHiC to other cell types and mouse embryonic data were still based on the previously trained model. Besides, we also trained DeepHiC on different chromosomes’ data in GM12878 dataset (in S5 Fig) only to show that our model is independent to the chromosomes we choose for training.

Besides, DeepHiC can also be trained on IMR90 and K562 datasets, which are other two deeply sequenced data from Rao et al., 2014. As shown in Supp Fig. 1a, 1b. We can see that they both converge and achieve nearly the same correlation as using GM12878 for training. Noting that the GM12878 dataset is the deepest sequenced Hi-C data (Supp Fig. 1c).

**Supp Fig. 1**

**Supp Fig. 1 Legend:** **a**) The SSIM scores in test set (chr15-chrX) are approaching nearly 0.9 when training DeepHiC on chr1-chr14 data in the K562 or IMR90 cell line. **b**) Pearson correlation in same dataset (chr15-chrX in IMR90) when using trained model from different cell type. **c**) Total read counts of 9 datasets (include 8 cell lines, the data of HeLa cell line is not available) from Rao et al., 2014.

2. A second key concern is why DeepHiC is able to perform better than a replicate experiment in GM12878. In theory, it should not be possible to perform better than a replicate experiment, as replicates differ from the original only in terms of sampling noise. It is imperative to understand why this is and report it in the text.

Below are a few hypotheses for how one could obtain better results with DeepHiC than from replicates.

- -  The sequencing depth of the two replicates is different. For instance, if replicate 1 used for training is sequenced deeper than replicate 2, then the model will predict with noise properties corresponding to the deeper sequencing depth, and the replicate experiment will have more limited power to detect the high-resolution features that DeepHiC was able to learn from the deeply sequenced replicate. To address this, the authors can subsample the more deeply sequenced replicate to the sequencing depth of the other one, then train a model where the high-resolution data input is the subsampled replicate, and then compare with the other replicate.
- -  There is actually a difference in a property of the data between the two replicates, and because DeepHiC learns this property, it is able to predict it better than the replicate experiment can. One such property may be the distance dependence of contact frequency. The authors can check if the distance dependence curves are different between the two replicates, and if they are, re-sample one replicate using the distance dependence curve of the other, and then perform the performance comparison. Other properties may be the restriction enzyme used, exact protocol, etc. which are listed in Rao et al. 2014, supplementary information in Supplemental Table S1.
- -  There are clonal differences between the two replicates which have structural variation that accounts for the differences in Hi-C profiles. For example, Rao et al., 2014 describe the following in the supplement: “Note that, to make our data maximally comparable to that of the ENCODE project, all of our GM12878 cells were obtained from Coriell’s “Expansion A”, from a lot set aside for ENCODE. The cells derive from two different batches. One of the biological replicates in our dilution experiment (labeled HIC034, br10) and one other biological replicate (HIC048, br 13) derive from the first batch (“Batch 1”, received at the Broad Institute on 3/11/2008); the rest of our biological replicates derive from the second batch (“Batch 2”, received at the Broad Institute on 5/13/2008).”

**Response**: Thank you for these comments, and we appreciate your professional analyses very much. According to Rao et al., 2014, The *GM12878* dataset refers to the ‘primary’ which contains HIC001-HIC018, and the *GM12878R* dataset refers to the ‘replicate’ which contains HIC019-HIC029. Their total intra-chromosome contact counts are 2,543,758,669 and 2,096,694,806, respectively. They are nearly in the same sequencing depth. Also, their libraries were prepared using the same restriction enzyme, exact protocol, and clonal batch.

At first, please allow us use a diagram to show four different ways of calculating correlations between replicates, as shown in Supp Fig. 2a, for addressing your concerns. The correlation of GM12878 and GM12878R in test set (chr15-chr22) are black dash lines in Supp Fig. 2b-e.

1. The Supp Fig. 2b was the figure we used in the manuscript. It showed the correlations between enhanced-Hi-C from downsampled data versus the original Hi-C in test set. These results revealed the ability of our model in fitting to the ground truth. Together with the same results in K562 and IMR90 cell lines, it revealed that our model could fit to the ground truth data when using its downsampled inputs. Thus, the black dash line in Supp Fig. 2b has no relationship with colored lines. Imaging that there is a worse/better replicate data, performance of fitting to the ground truth should not be influenced by the replicate. In conclusion, the black dash line in this figure doesn’t make sense, colored lines indicated that our model’s performances decayed when fitting to the ground truth along with the downsampling ratio increased.

2. The Supp Fig. 2c-d are the circumstances that happen in reality. For instance, we had a deeply sequenced data GM12878R, then we sequenced a replicate in low sequencing depth, which could be downsampled-GM12878. By DeepHiC imputation, the correlation of enhanced data is as good as the real replicate, which is shown in Supp Fig. 2c, or in Supp Fig. 2d for the opposite. Further, if we had two low sequenced Hi-C data, enhancement on both of them could also improve the correlation between them, as shown in Supp Fig. 2e. The slight superiorities may suggest that our model is capable of denoising, which was also claimed in Goodfellow et al., 2014 (the original GAN paper). In revised manuscript, we replaced Fig. 1d with Supp Fig. 2c.

**Supp Fig. 2**

**Supp Fig. 2 Legend: a)** diagram of four ways to calculated correlations between replicates shown in **b-e**. **b)** is the Figure 1d in old manuscript. **c-e)** correlations between ‘primary’ and ‘replicate’ data in predicted/original/downsampled datasets in the test set (chr15-chr22) in different ways according to the diagram in **a**.

3. Related to the above comment, how variable are the predictions as a function of restriction enzyme used?

**Response**: Thank you for the comment. Both the high-resolution *in situ* Hi-C library from Rao et al., 2014 and the mouse embryonic *in situ* Hi-C library from Wei Xie Lab were prepared using the MboI enzyme. We test DeepHiC (no re-training) on Hi-C data produced using 6-cutter restriction enzyme (dilution Hi-C, HindIII&NcoI, HIC034-037) and show the performances in Supp Fig. 3. We can see that DeepHiC could also make prediction on 6-cutter digested Hi-C data and achieved the same improvements as predicting on data prepared using MboI.

**Supp Fig. 3**

**Supp Fig. 3 Legend**: Performances of correlation and SSIM scores when apply DeepHiC to GM12878 HindIII(&Ncol) Hi-C data.

4. There are a few additional benchmarks that would strengthen the paper, specifically showing the specificity of predictions across different conditions. A general concern for methods predicting HiC data is that if a model predicts an average HiC profile across cell types, it will look like a strong model since most TADs are constant between different cell types. Thus, it would be instructive to show that the model is able to pick up on cell type specific differences, as defined from the high-resolution data, for example changes in TAD boundary locations or appearance/disappearance of TADs. Conceptually, DeepHiC should indeed correctly identify cell type specific features, since during training it only sees one cell type, and thus does not “know” how features such as TADs vary between cell types. However, it would be useful to confirm this analysis to the paper, as it would increase the confidence of the community in the method.

**Response**: Thank you for your suggestion. We agree with you that we must make sure that our model does not predict an average Hi-C profile across cell types. We used a heatmap of correlations between different cell types to demonstrate that our model also preserves the specificities of cell types. We calculated the correlations across 9 datasets (8 cell lines) from Rao et al., 2014 (black numbers in Supp Fig. 4). Then, downsampled them to 1/16 ratio and make prediction on downsampled data using model trained on GM12878. Correlations between real cell-A data vs. predicted cell-B data are magenta numbers in Supp Fig. 4. Correlations across cell types suggests that our model preserves the specificities among cell types. Pearson correlation of black numbers and magenta numbers (exclude the diagonal) is 0.99 with p-value=1.44e-26). We add this result to S17 Fig.

**Supp Fig. 4**

**Supp Fig. 4 Legend:** correlations between Hi-C data across different cell types, black numbers represent correlations between two real data. Magenta numbers represent correlations between real data and predicted data (1/16 downsampled). Median correlation: median value of Pearson correlations from 50 kb to 1 Mb genomic distance.

5. The authors show the performance across 4 cell types, even though Rao et al., 2014 have profiled 9 cell types. Please report the performance across all cell types in that study.

**Response**: Thank you for your suggestion. The Hela-S3 cell line data is not available. We applied the downsampling (1/16) procedure and applied DeepHiC them in other 5 cell types: KBM7, NHEK, HUVEC, HMEC, and CH12-LX (mouse). The results are shown in Supp Fig. 5. We added this result to S18 Fig.

**Supp Fig. 5**

**Supp Fig. 5 Legend:** The performances of DeepHiC in KBM7, NHEK, HUVEC, HMEC, and CH12-LX cell lines, downsampling ratio is 1/16.

6. Conceptually, DeepHiC should generalize across species. Please show if this is the case.

**Response:** Thank you for your comment. Our model could generalize across species. In the last result section. We applied DeepHiC to the mouse embryonic Hi-C data which obtained from Wei Xie Lab. We still used the previously trained model which trained on chromosome 1-14 in *GM12878* dataset. Results on mouse embryonic Hi-C data demonstrated that our model may be applied across species. And in **Response 5,** CH12-LX is a mouse cell line. Results on this dataset demonstrated that our model could generalize across species.

7. The authors mention that they have implemented a parallelized version of Fit-Hi-C. Please confirm that the re-implementation and the original code produce the same results on a test dataset.

**Response**: Thank you for your suggestion. We added a test demo to the GitHub repository (<https://github.com/omegahh/pFitHiC>) and putted the test results in the following for convenience. P-values and q-values of every bin pairs are exactly same between original FitHiC (in x-axis) and my code (in y-axis).

**Supp Fig. 6**

**Supp Fig. 6 Legend:** Validation of our re-implementation of Fit-Hi-C

8. When comparing DeepHiC with Fit-Hi-C called interactions, I am not sure I understand the procedure. Fit-Hi-C requires the user to provide counts for each interaction. Please clarify how the output from the neural network is converted to counts, or alternatively what inputs are provided to Fit-Hi-C.

**Response**: Thank you for your comment. We converted the output to integral counts for Fit-Hi-C input. Specifically, we convert the output values to integer by multiplying them with 255 and converting to integer type. It makes sense that DeepHiC is trained to fit the contact map ranges from 0-255 before min-max normalization. And it can be easily verified by comparing the sum value between high-resolution contact map and predicted contact map in other datasets. For example, after max-min normalization, the sum value of the former is 2176479.15, and the latter is 2259598.32, which is 1.03 times to the former, in K562 dataset. We have added this detail to the revised manuscript.

9. It is not clear how the authors set a cutoff for significant interactions called by Fit-Hi-C. The authors write “We kept the predicted significant interactions (q-value < 0.5-percentile) for genomic distances from 20 kb to 1 Mb for further comparative analysis.”. Does this mean that they take the top 0.5% of interactions, as sorted by q-value and call those significant? Or that they set a threshold on the q-value of 0.05? To my understanding, a concrete threshold should be set on the q-value, rather than selecting the top x% interactions. If we consider a dataset with reads uniformly distributed in the contact map, then there are no significant interactions, but the approach of selecting the top x percentile would produce a set of interactions. Please either modify the procedure to use a concrete q-value threshold, or clarify in the text if this is what was done.

**Response**: Thank you for the comment. It was the top 0.5% of q-values used. Through top 0.5% of q-values are all far smaller than 0.001, we revised it to a concrete threshold. It is 1e-6, which also used in HiCPlus paper. This change involves the Fig. 4a, 4c and Fig S21, as well as Fig. 6b, 6c, and 6d in last result section. We have re-implemented the evaluations and revised the manuscripts.

10. It would be useful to translate the sequencing depth from fractions (e.g. 1/16) to the actual sequencing depth for the dataset, so that readers/users can assess the amount of sequencing required to use DeepHiC. Please specify in the discussion the lower bound of reads required for DeepHiC performance to be high.

**Response**: Thank you for your suggestion. Translating the downsampling factors to read coverage or data distribution is useful for researchers/users. We add a guide in Supplementary Note (S2 Note). Considering that there is no concrete criterion of 10-kb Hi-C data, the strategy of choosing model should be related to the training data we used. The detailed answer is listed in the following:

1. **Users could choose the model according to the distribution of their Hi-C matrix**, especially focus on the values of top 95-99.9 percentiles (in all non-zero values). As shown in Supp Fig. 7a, for different downsampled data, the cutoff values for low-coverage input (*-lrc* parameter for our code in GitHub) were close to the 99.9 percentile values in our trained data. For a low-coverage data, user should use the model where their 99.9-percentile value is close to or lower than the cutoff value of chosen model. A caveat is that user should not use 25 as a cutoff value while the 99.9 percentile of their data is 100. Therefore, the model for 1/100 downsampled data is not always the best choice.

2. **If user has a Hi-C data which is latently in 1/16 to GM12878 data, it should be better to use the model trained on 1/16 downsampled data.** We downsampled GM12878 data to 1/25, 1/36 ratio, but still using the model trained in 1/16 downsampled data. These results have been shown in Fig. S17. Our model still worked well but performance decayed.

3. **Another important metric is the non-sparsity of input data must greater than ~10%.** Since our low-coverage input is still binned at 10-kb, the non-sparsity (#non-zeros/#all-pairs) of input decreased when the downsampled factor increased, as shown in Supp Fig. 7b. That means for 1/100 downsampled data (non-sparsity=9%), our model imputes ~75% cells from zeros to fit the original data (non-sparsity=84%). A lower non-sparsity of input data reaches the lower bound of our current model. Thus, prediction in 1/100 downsampled data of K562 and IMR90 didn’t worked because their 1/100 downsampled data are in lower non-sparsity (Supp Fig. 7c)

**Supp Fig. 7**

**Supp Fig. 7 Legend: a)** The trend of top 95-99.9 percentile non-zero value in GM12878 downsampled data. The cutoff value is used for model input. **b)** The non-sparsity of training data in different downsampling factors. **c)** The non-sparsity of IMR90 and K562 data in different downsampling factors.

11. In Fig S6, why are SSIM scores so bad across chromosomes for the replicates? And why are they so variable? I wonder if there is a technical artifact accounting for this.

**Response**: Thank you for your comments. We did the following analyses which suggest different level of similarity across chromosomes between replicates may cause variable SSIM rather than a technical artifact. First, the old Fig S6 (now Fig S8) is calculated in the same manner with the Supp Fig. 2b. We replaced it with the same manner with Supp Fig. 2c, while it contains chromosome 1-14 (the training set), as shown in Supp Fig. 8a. Second, for analysis the reason of the variation. We found that correlations for each chromosome were also variable, and had similar trends with SSIM score for real high-resolution replicates, as shown in Supp Fig. 8b.

**Supp Fig. 8**

**Supp Fig. 8 Legend: a)** re-calculation results of SSIM scores between replicates. **b)** comparison of SSIM score and correlations between the real high-resolution replicates for each chromosome. whiskers: Q1-1.5*IQR, Q3+1.5*IQR, orange line: median, box: Q1 and Q3

12. The biological validation in Figure 6. The authors compute Fit-Hi-C significant interactions and measure the performance as the fraction of significantly called Fit-Hi-C interactions that anchor at i) promoters or ii) at ATAC-seq peaks. It is not clear to me that this is a valid biology-related performance metric. If DeepHiC were to connect all promoters and all ATAC-seq peaks, it would get a high score by this metric without being biologically sound. To alleviate my concern, the authors do check whether this assumption holds true in deeply sequenced HiC data, which is the case, compared to a shuffled control. In this case, the authors can suggest that the distribution of Fit-HiC calls is more similar to what would be obtained from a high-resolution dataset. While this makes the case that DeepHiC is more similar to deeply sequenced HiC data, it still in my mind does not say that the loops found using DeepHiC as an input are “better” biologically than those from the lowly sequenced data. Thus, the authors should clarify the wording when describing this section.

Related to this, in the introduction, the sentence “ In this study, we applied DeepHiC to Hi-C data in mouse embryonic development and demonstrated that, compared with the original low-resolution Hi-C data, DeepHiC-enhanced Hi-C data provides more interpretable results for the identification for chromatin loops.” should be revised. More interpretable does not necessarily mean correct.

**Response**: We thank the reviewer for the careful review and the helpful advises. We compared the Fit-Hi-C interaction and shuffled interaction in terms of their propensity to anchor at promoters or open chromatin regions (GM12878 DNase-seq peaks retrieved from ENCODE, accession number: ENCSR000EMT) in GM12878 cell line. As expected, we found a significant higher proportion of Fit-Hi-C interactions at both promoter and open chromatin regions, consistent with the results in Figure 6. We have added these analyses in the revised manuscript.

**Supp Fig. 9**

**Supp Fig. 9 Legend: Properties of Fit-Hi-C identified significant interaction in high-resolution Hi-C data of GM12878 cell line. a)** Fraction of significant interactions of which both connected loci contain DNase-seq signal peaks. Error bar: stardar deviation. **b)** Fraction of significant interactions anchor loci which are interested with gene promoters. Error bar: standard deviation.

Also, we changed the description in the revised manuscript from “In this study, we applied DeepHiC to Hi-C data in mouse embryonic development and demonstrated that, compared with the original low-resolution Hi-C data, DeepHiC-enhanced Hi-C data provides more interpretable results for the identification for chromatin loops.” To “In this study, we applied DeepHiC to Hi-C data in mouse embryonic development and demonstrated that, compared with the original low-resolution Hi-C data, DeepHiC-enhanced Hi-C data empowered identification of loops more similar to deeply sequenced Hi-C data.”. We also changed description in the section accordingly.

13. A good validation for loops, as defined in Rao et al., 2014 would be to look at loops which are different across cell types. Since these loops are anchored by binding of CTCF, one would expect that when CTCF is absent in a cell type, the loop should be absent at a given site, and be present when CTCF is bound. By combining CTCF binding information and HiC the authors can check whether DeepHiC is able to correctly identify these differential loops.

**Response**: Thank you for the suggestion. We used the CTCF targeted ChIP-seq data from ENCODE (Snyder Lab, Stanford) in GM12878, IMR90, and K562 cell line. We used the processed bed file (optimal IDR thresholded peaks). We got the cell-line-specific peaks, and assigned them to 10 kb bins. For DeepHiC-enhanced Hi-C data in GM12878R, IMR90, K562 cell line, using q-value<1e-6 for Fit-Hi-C threshold, we got the cell-line-specific significant interactions (SI). As shown in Supp Fig. 10, For SIs binding with CTCF, they are enriched in their own specific CTCFs. These results suggest that DeepHiC is able to correctly identify these differential loops.

**Supp Fig. 10**

**Supp Fig. 10 Legend:** Enrichment of cell-line-specific Fit-Hi-C predicted significant interactions with binding the cell-line-specific CTCFs.

14. The choice of applying DeepHiC to mouse early embryonic growth is very good, given that there is a limitation on the number of cells present, rather than just an economic motivation for low-resolution data.

**Response**: Thank you for the comment.

15. For the ChIA-PET analysis in the section “Chromatin loops in high-resolution Hi-C were accurately recovered from DeepHiC-enhanced matrices”, the authors describe their selection of the negative sets as follows: “As for negatives, we randomly selected the same number of loci pairs that were not predicted to be interacting pairs by ChIA-PET (10 repeats).”. It would be more accurate to use a set of interaction pairs that are matched to the positive set in terms of distance between them.

**Response**: Thank you for the suggestion. We re-implemented the ROC analysis according to your suggestion and updated the Figure 4e in our manuscript. The frequency of ChIA-PET pairs in terms of genomic distance is shown in Supp Fig. 11.

**Supp Fig. 11**

**Supp Fig. 11 Legend:** Frequency of pairs along with genomic distance from 20 kb to 1.2 Mb in the ChIA-PET data we used in ROC analysis.

16. “We mainly focused on the 8-cell stage and beyond because Hi-C data from earlier stages only demonstrate weak TADs and depleted distal chromatin interactions”. Please show the analysis for these as well. If there are no TADs, then we would expect to see fewer interactions. Also, the structure is more different than what the model has seen in the training set, and thus it will be interesting to see the performance of DeepHiC.

**Response**: Thank you for the detailed review. We have updated results in 2-cell early and 2-cell late stages in the revised manuscript. The results show that, for both original Hi-C data and DeepHiC enhanced data, the Fit-Hi-C interactions are less likely to anchor at ATAC-seq peaks and promoters in 2-cell early stage and 2-cell late stage than in 8-cell stage. The fraction of interaction identified with DeepHiC enhanced data are less likely to anchor at promoter and ATAC-seq peaks than those identified with Hi-C data in 2-cell late stage, while the shuffled interactions corresponds to the highest proportion. We think that these results may be due to the depletion of chromatin loops in 2-cell stages.

17. What is the lowest size of genomic bin that can be used with DeepHiC? Does it work well at 5kb resolution? What about 1kb? Please add a figure showing performance as a function of resolution of the data at which the training is done.

**Response**: Thank you for the suggestion. We trained DeepHiC using the GM12878 data at 5 kb and 1 kb, and found that it could be successfully trained in 5 kb, while failed in 1 kb, as shown in Supp Fig. 12. Noting that binning at 5 kb would increase 4x samples, binning at 1 kb would increase 100x samples. That means the training time for 5 kb and 1 kb are 4x and 100x to training for 10 kb (~9 hour, 200 epochs).

**Supp Fig.12**

**Supp Fig. 12 Legend:** **a)** SSIM scores in test set (chr15-22) when training with data binned at 5-kb. **b)** SSIM scores in test set (chr15-22) when training with data binned at 1-kb. Training using 1-kb data failed using current training scheme.

18. The repository at https://github.com/omegahh/DeepHiC is very well written. It would further benefit from showing an example tutorial for running the model on a dataset that is not in the format of Rao et al., 2014. For instance, the authors could describe clearly the formats in which they expect the data to be, and describe in more detail what the different arguments are for the scripts being used.

**Response**: Thank you for the helpful advice. We have updated the README file in the GitHub repository, and uploaded a script of converting HiC-Pro file to our model input.

19. The exciting performance of DeepHiC suggests it may do just as well predicting the rest of the contact matrix off the diagonal (i.e related to compartments). A future direction (out of the scope of the current study, perhaps) is to use the same model, or a separately trained model to predict the rest of the contact map, in order to obtain better resolution for the borders of A/B compartments, as well as for inter-chromosomal contacts.

**Response**: Thank you for the helpful suggestion. We will focus on improving the performance on predicting borders of A/B compartments as well as inter-chromosomal contacts in our future research.

20. It would be useful to check how distance dependence differences between datasets are preserved by DeepHiC. For instance, during the cell cycle, the scaling of contact frequency changes dramatically. It would be useful to confirm that DeepHiC preserves this difference.

**Response**: We thank the reviewer for helpful advice. We check the distance dependence in different cell types. The results suggest DeepHiC preserves differences in distance dependence.

**Supp Fig. 13**

**Supp Fig. 13 Legend:** Distance dependence of original, DeepHiC-enhanced, and downsampled Hi-C data in different cell types. Downsampling ratio: 1/16

21. Figure 3c shows that DeepHiC outperforms other methods when stratified by distance. It appears that there is a large gap in performance between DeepHiC and HiCNN at very small distances (<20kb), and that after 20kb, the performances between the two methods become much more similar. It would be useful to understand why DeepHiC performs better at low distances.

**Response**: Thank you for the comment. The plotting unit is at 10 Kb in Figure 3c and the range corresponds to about 50 Kb to 200Kb. A potential reason why DeepHiC performs better at low distances may related to the large amount of information entropy in these regions. The original GAN paper (Goodfellow et al., 2014) said that “Another advantage of adversarial networks is that they can represent very sharp, even degenerate distributions”. We calculated the entropies of every 9x9 chunks grouped by the genomic distances of their centres, which are from 50 kb to 800 kb with step 50kb, as shown in Supp Fig. 14. The entropies decay along with the genomic distance increases.

**Supp Fig. 14**

**Supp Fig. 14 Legend:** Entropies of all 9x9 chunks where their centres are located in (x_i, x_j), where j minus i is the genomic distance, whiskers: Q1-1.5*IQR, Q3+1.5*IQR, orange line: median, box: Q1 and Q3

**Minor comments**

22. Be precise when using the word loops. “Loops” has been used to describe multiple different features of Hi-C maps. Specifically, some papers call chromatin contacts that significantly deviate from the expected contact frequency as loops, whereas other papers understand loops to be regions of enriched contact frequency relative to the surrounding chromatin (e.g. as defined in Rao et al., 2014).

For example, in the section “Application of DeepHiC improves identification of chromatin loops in mouse early embryonic developmental stages”, the word loops is used to mean both definitions: “Chromatin loops regulate spatial enhancer-promoter contacts and are relevant to domain formation [4, 53], and anchors of chromatin loops co-localize with open chromatin regions including insulators, enhancers, and promoters.”. Please revise to make the text more clear.

**Response**: Thank you for the comment. We replaced ‘loops’ with ‘significant interactions’ in many cases in the revised manuscripts to avoid the confusion.

23. I would modify the name of DeepHiC to include a reference to the fact that the model is GAN. However, this is just a suggestion and I leave it to the authors to make the final decision.

**Response**: Thank you for the kind suggestion. DeepHiC is named with two means: a **deep** learning model **deepen** the Hi-C data sequencing depth.

24. The authors mention that “Because of the high cost of sequencing, most available Hi-C datasets have relatively low resolution, which limits their application in studies of genomic regulatory elements”. Define low resolution in the text and give a number to demonstrate the limited applicability for studying regulatory elements.

**Response**: Thank you for the comment. Sorry for the unclear description. We revised this sentence to “Because of the high cost of sequencing, most available Hi-C datasets have relatively low resolution, such as 25 kb or 40 kb [Zhang et al. 10.1038/s41467-018-03113-2].”. 40-kb resolution is low resolution we referred. Besides, it should be more accurate that high-resolution can be used to recognize more refined structures, so we deleted “which limits their application in studies of genomic regulatory elements”.

25. “Low resolution data may be less effective for detecting large-scale genomic patterns such as A/B compartments”. Compartments are frequently called at 1Mb resolution, so I would say that compared to other 3D genome features, this is actually effectively estimated even with low coverage.

**Response**: Thank you for the comment. It should be ‘sufficient instead of ‘less effective’. We have revised the manuscript.

26. I can only see the figures in low resolution, which makes it difficult to properly visually assess Figures 2, 4.

**Response**: Sorry for the inconvenience. The uploaded figures are all in 300x300 dpi. We can send them to you by e-mail if they are still blurred after submission.

27. Please clarify the normalization scheme needed for the inputs to DeepHiC (sqrtvc, KR, etc.). Ideally DeepHiC is applied to normalized data, rather than raw data that is biased by uneven coverage of genomic bins.

**Response**: Thank you for the comment. We used the KR normalization scheme for normalizing data. We revised the manuscript accordingly.

28. In Figure S17, add replicate as another benchmark.

**Response**: Thank you for your suggestion. The updated Figure (Fig. S21) have added the performance of replicate, also shown in the Supp Fig. 15

**Supp Fig. 15**

29. The authors refer to downsampled data as low-resolution data. While it is true that having fewer reads warrants using a lower resolution, the authors use the low-resolution data binned at a high resolution which can be confusing to the reader. To avoid this confusion, I would suggest to use “low coverage” (which the authors use sometimes) rather than “low resolution”.

**Response**: Thank you for your suggestion. We revised the manuscript according to your suggestion.

30. For Boost-HiC, did other parameters of alpha result in better performance?

**Response**: Thank you for your comment. We tried several different alpha ranges from 0.1 to 0.3 with step 0.1 on chromosome 22, and found that there are no obvious improvements. An *alpha value close to 0.2* is the recommended according to the author (<https://github.com/LeopoldC/Boost-HiC/issues/1>). On April 9, the author changed it and said that 0.24 is the best value (<https://github.com/LeopoldC/Boost-HiC/>, commit: 396c5df). Importantly, the implementation of Boost-HiC is O(n5) in time complexity (also see <https://github.com/LeopoldC/Boost-HiC/issues/1>). In chromosome 22, It costs ~7.7 minutes in a Xeon E5-2682 v4 @ 2.5GHz CPU. It means that implementation on chromosome 1 will cost ~379 days. We ran Boost-HiC by centromeric arm on human dataset as suggested by the author. It still costs us several weeks on our HPC cluster.

31. The sentence “DeepHiC can be used to enhance the resolution of existing time-resolved Hi-C data obtained through early embryonic growth. These data are prone to low resolution due to limited cell population.” should include in parentheses the resolution that was used.

**Response**: Thank you. The author (Wei Xie *et al.,* doi:10.1038/nature23263) of mouse embryonic growth data binned them in 40-kb resolution. We have added it to this sentence.

32. The A/B compartment analysis described in “DeepHiC outperformed other methods in terms of genome-wide similarity”” would be strengthened if it were summarized with a statistic related to figure S14, for instance the percent of genomic regions assigned to the same compartment between two methods, or the correlation between compartment scores.

Also, Figure S14 is incorrectly referenced as Figure S4 in the relevant paragraph - please correct this.

**Response**: Thank you for your suggestions. We have corrected the figure number (Fig. S16 in revised manuscript) and added the quantitative results to the legend of Fig. S16. Correlations of DeepHiC and BoostHiC versus experimental are 0.952 and 0.966, respectively. And Jaccard indices of DeepHiC and BoostHiC versus experimental are 0.906/0.896 and 0.908/0.916 for each A/B compartment, respectively.

33. Please add a sentence in the methods section describing how the ChIA-PET data were processed, in addition to the currently present description of where they were downloaded from. How were significant interactions defined? And how were these “assigned” to Hi-C bins in later analyses?

**Response**: Thank you for the suggestion. Sorry for the unclear description. We used the processed bed file from ENCODE. Specifically, we downloaded the processed data for ENCODE (filename is ENCFF001THV.bed.gz from experiment ENCSR000CAC). It also can be downloaded from UCSC browser (track name: wgEncodeEH002075). Second, to assign chromosome regions to Hi-C bins. We made all overlapped bins be positive bins. For example, chr1: 954000-973000 is assigned to three Hi-C bins: 950000-960000, 960000-970000, 970000-980000 at 10-kb resolution. We revised manuscript accordingly.

34. I recommend a careful read through the paper to correct any typos or other small errors such as incomplete sentences.

**Response**: Thank you for your suggestions. We have carefully checked the manuscripts several times and corrected errors, with assistance of a colleague.

35. For Figure 2, please add numbers of the colorbars to denote the upper and lower bounds. Currently, these are labelled as “high” and “low”.

**Response**: Thank you for the comment. We have added numbers to them in Figure 2.

**Future directions outside the scope of the current study**

A direction for future work would be to apply such a model to very high-resolution 3D genome data such as MicroC-XL.

Another future direction will be to check the applicability of DeepHiC to single cell HiC data.

Finally, since DeepHiC’s impact is the largest in cases where it is challenging to collect deeply sequenced HiC data, it would be interesting to check how DeepHiC enhances allele-specific contacts (e.g. as described and computed in Rao et al., 2014). Allele-specific contact maps rely on SNPs to annotate the allele for the reads in the dataset, resulting in severely diminished numbers of reads ultimately being used.

**Response**: Thank you very much for these helpful and professional suggestions. Train DeepHiC on Micro-XL data is an excellent idea. We will focus on it in the future. Also, for enhancing single cell Hi-C and allele-specific Hi-C, we are going to add more tests on them.

**Response to reviewer #3**

*Comments to the Authors:*

Hong, Jiang et al in the manuscript "DeepHiC: A Generative Adversarial Network for Enhancing Hi-C Data Resolution" implement a generative adversarial network that interpolates low resolution HiC maps to produce higher-resolution maps. Their implementation offers some quantitative improvements over previously published methods. Unfortunately, the manuscript does not offer sufficient biological or methodological insight to justify publication in this journal. Their success in interpolating the data confirm the low amount of information entropy present in large HiC data sets, as it was previously shown. As a consequence, the manuscript does not seem of high importance to researchers in the field. I suggest submitting the manuscript to some more technical journal.

**Response**: Thank the reviewer for reviewing our work. We don’t agree with the reviewer #3. Due to sequencing cost or experimental limitation, the resolution of most Hi-C data is coarse, resulting in a loss of information and biological interpretability. In this study, we developed a computational model to increase the effective sequencing depth of Hi-C data. We demonstrated that our model substantially outperforms previous methods in Hi-C data resolution enhancement, especially in boosting accuracy in chromatin loops identification and TADs detection and applied it to mouse embryonic Hi-C data.

1. In methodology, we take advantage of GAN framework in representing data with sharp or degenerate distributions to enhancing Hi-C data in short-range contacts. Also, we integrated multiple loss functions to encourage both good reconstruction error and structural similarity with the high-resolution data.

2. In biology, we applied our model to mouse embryonic Hi-C data, which is in low-coverage due the limitation of number of cells present. Enhanced mouse embryonic Hi-C data by our model achieves better results in identification of chromatin loops, which may facilitate the studies on gene regulation of mouse embryonic development.

Above all, we think our work is suitable for PLoS Computational Biology
